# Amino acid metabolism in health and disease

**DOI:** 10.1038/s41392-023-01569-3

**Published:** 2023-09-13

**Authors:** Zhe-Nan Ling, Yi-Fan Jiang, Jun-Nan Ru, Jia-Hua Lu, Bo Ding, Jian Wu

**Affiliations:** 1 Division of Hepatobiliary and Pancreatic Surgery, Department of Surgery, The First Affiliated Hospital, Zhejiang University School of Medicine, 79 Qingchun Road, Hangzhou, Zhejiang Province 310003 P.R. China; 2 NHC Key Laboratory of Combined Multi-organ Transplantation, Hangzhou, Zhejiang Province P.R. China; 3 Key Laboratory of the Diagnosis and Treatment of Organ Transplantation, Research Unit of Collaborative Diagnosis and Treatment For Hepatobiliary and Pancreatic Cancer, Chinese Academy of Medical Sciences (2019RU019), Hangzhou, Zhejiang Province P.R. China; 4 Key Laboratory of Organ Transplantation, Research Center for Diagnosis and Treatment of Hepatobiliary Diseases, Hangzhou, Zhejiang Province P.R. China

**Keywords:** Cancer metabolism, Cancer metabolism

## Abstract

Amino acids are the building blocks of protein synthesis. They are structural elements and energy sources of cells necessary for normal cell growth, differentiation and function. Amino acid metabolism disorders have been linked with a number of pathological conditions, including metabolic diseases, cardiovascular diseases, immune diseases, and cancer. In the case of tumors, alterations in amino acid metabolism can be used not only as clinical indicators of cancer progression but also as therapeutic strategies. Since the growth and development of tumors depend on the intake of foreign amino acids, more and more studies have targeted the metabolism of tumor-related amino acids to selectively kill tumor cells. Furthermore, immune-related studies have confirmed that amino acid metabolism regulates the function of effector T cells and regulatory T cells, affecting the function of immune cells. Therefore, studying amino acid metabolism associated with disease and identifying targets in amino acid metabolic pathways may be helpful for disease treatment. This article mainly focuses on the research of amino acid metabolism in tumor-oriented diseases, and reviews the research and clinical research progress of metabolic diseases, cardiovascular diseases and immune-related diseases related to amino acid metabolism, in order to provide theoretical basis for targeted therapy of amino acid metabolism.

## Introduction

The primary function of amino acids is to act as the monomer unit in protein synthesis and as substrates for biosynthetic reactions.^1,2^ Amino acid metabolism disorders have been linked to the progression of various diseases. For example, the deletion of tumor suppressor genes, such as PTEN and P53 or the activation of tumor genes, such as c-Myc and Ras, may induce changes in nutrient supply, metabolic enzymes, metabolic requirements and many other metabolic characteristics. Therapeutically targeting tumor cell metabolism have been proven effective with fewer side effects compared to some conventional treatments.^3,4^ Moreover, therapies targeting essential amino acids, such as dietary methionine restriction has been shown to extend lifespan in mice and rats.^5–8^ Tumors likely rely on external supply of nonessential amino acids.^9^ Therefore, restriction of these amino acids can inhibit tumor growth, demonstrating the importance of amino acid metabolism. Besides its role in cancer, amino acid metabolism has been reported as an important participant in the development of metabolic diseases such as diabetes and obesity, as well as cardiovascular diseases, autoimmune diseases and neurological diseases.^10–20^ Herein, we discussed the metabolism of amino acids in health and disease and the potential clinical application of amino acid metabolism in treating cancer and other diseases.

## Overview of amino acid metabolism

Amino acids are organic compounds containing amino and carboxyl groups, which can be divided into α-, β-, γ-, δ- amino acids according to the position of the functional groups of the core structure, the most important of which are the 22 alpha-amino acids that make up proteins and 20 of these amino acids are involved in protein synthesis. Amino acids are involved in biosynthesis, neurotic transmission, and other life processes.^1,2^ Peptide bonds link amino acids to form polypeptide chains, which undergo post-translational modifications and sometimes combine with other polypeptide chains to form proteins. Among amino acids that make up proteins, nine cannot be synthesized from other compounds and must be obtained from food; these are also essential amino acids.^21,22^ When amino acids are ingested by the human body from food, in addition to being used for protein and other biomolecular synthesis, they can also be oxidized to urea and carbon dioxide as energy sources through oxidative pathways.^23^

**Fig. 1:**
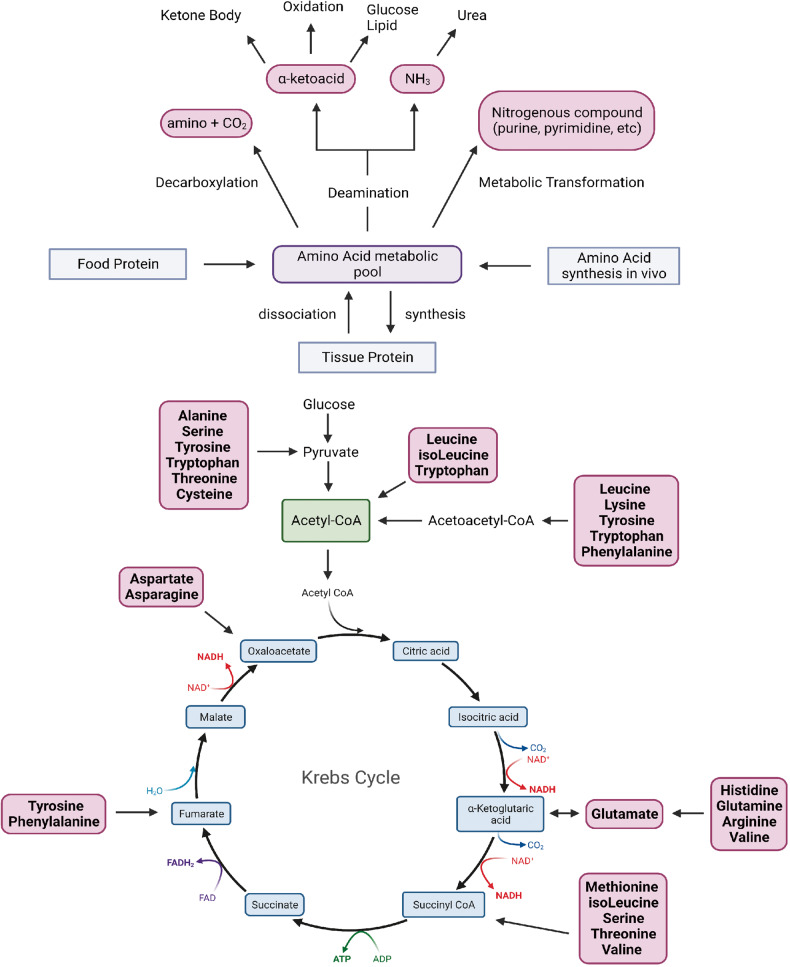
Overview of amino acid metabolism. The human body can obtain amino acids through food digestion and absorption, tissue decomposition, internal synthesis of three ways. Amino acids in amino acid metabolism pool can be deacidified to produce amino and carbon dioxide. Or participate in the synthesis of purine, pyrimidine and other nitrogenous compounds in the transformation of metabolites; Or deamination produces α-ketoacid and NH3. According to different enzymes and pathways, α-ketoacid can produce keto bodies, or participate in oxidative energy supply or sugar and lipid synthesis; NH3 enters the urea cycle. Created with BioRender.com

The oxidation pathway begins with aminotransferase-mediated deamination and transfers the amino group to alpha-ketoglutaric acid to form glutamate to enter the urea cycle. Another product, keto acid, enters the citric acid cycle to provide energy for life activities (Fig. 1).^24^ The uptake of amino acids by cells or organelles requires the participation of amino acid transporters (AATs). Different amino acids depend on specific AATs, but amino acids and transporters are not one-to-one matched. Multiple AATs can transport an amino acid, and the same transporter can also transport multiple substrates. In addition to serving as a channel for amino acids to enter and exit the cell, AATs also function as a probe for sensing amino acid levels and as an initiator of nutritional signals.^25,26^ According to the diversity of structure and function, AATs can be divided into different families, in which the solute carrier (SLC) superfamily accounts for about 20% of all membrane proteins encoded by the human genome and is the largest superfamily of membrane transporters.^27^ According to substrate specificity, AATs can be divided into neutral, basic, and acidic categories, and further subcategories, including sodium-dependent and sodium-independent types. Mechanistically, because amino acid concentrations in the intracellular fluid are generally higher than those in the extracellular fluid in mammalian cells, including humans, AATs transport amino acids through ion conjugation or amino acid exchange to produce sodium ions. Hydrogen or chloride cotransporters and potassium reverse transporters maintain intracellular and extracellular Na^+^ and K^+^ concentration gradients through Na^+^/K^+^-ATP pumps.^28^ For the specific classification, function, and mechanism of AATs in the human body, we invite readers to review the following literature.^27,29,30^

In addition to being components of peptides and proteins, amino acids are involved in key pathways that maintain cell growth, metabolism, and immunity.^31–35^ For example, the mammalian target of rapamycin (mTOR) signaling pathway is a major mechanism that regulates protein synthesis.^36^ The mTOR system contains rapamycin-sensitive complex 1 (mTORC1) and rapamycin-insensitive complex 2 (mTORC2). mTORC1 is activated by glutamine (Gln), arginine (Arg), and Leucine (Leu), and activates protein synthesis by phosphorylation of eIF4E binding protein 1 (4E-BP1) and ribosomal protein S6 kinase 1 (S6K1).^37–39^ Furthermore, alanine (Ala) can regulate gluconeogenesis and glycolysis by inhibiting alanine kinase, thereby maintaining the amount of glucose produced by the starved liver.^38^ Arginine regulates the active state of the urea cycle by acting as an allosteric activator of N-acetyl glutamate synthetase (a mitochondrial enzyme that converts glutamate and acetyl CoA to N-acetyl glutamate).^40^ In terms of immunity, amino acids are involved in immune cell proliferation, differentiation and functional activation. For example, T cell activation upregulates a variety of amino acid transporters, including SLC7A5, and deletion of SLC7A5 leads to activation of the mTOR signaling pathway and upregulation of the transcription factor MYC to inhibit T cell proliferation.^41^ When T cells are deprived of Trp and Arg, activated T cells cannot enter the S phase, which proves that Trp and Arg are key substances for T cells to enter the cell cycle. Moreover, the depletion of Leu and isoleucine (iLe) induces T cells to enter the S-G1 phase, which then stops dividing and expires.^42–44^

In summary, amino acids are essential organic compounds for life support, as raw materials for biosynthesis and as a source of energy for life activities. The cellular uptake of amino acids requires the involvement of AATs. Transporters serve as the entry and exit channels of amino acids and act as probes for sensing amino acid concentrations and promoters of nutritional signals. In addition to being a raw material for biomass and an energy source, amino acids are also involved in key pathways in terms of cell growth, metabolism and immunity.

## Branched-chain amino acids (BCAAs)

### BCAAs metabolism

**Fig. 2:**
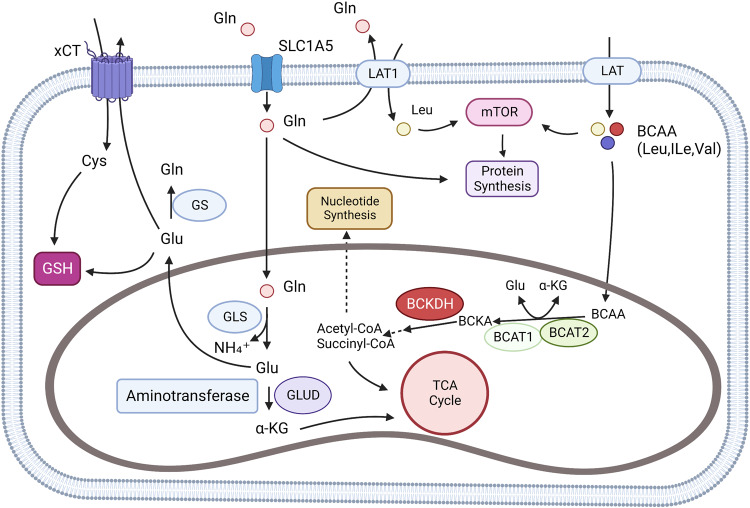
Glutamine and BCAA metabolism. BCAAs can be absorbed by the cell through L-type amino acid transporter (LATs), and L-type amino acid transporter 1(LAT1) can also exchange intracellular glutamine with extracellular leucine. In cells, BCAAs are catalyzed to formα-ketoisocaproate (KIC), α-ketoisovalerate (KIV), and α-keto-β-methylvalerate (KMV). The three substances are collectively known as branched alpha-ketoacids (BCKAs). Further, BCKAs produce acetyl-CoA through an irreversible rate-limiting reaction catalyzed by branched alpha-ketoate dehydrogenase (BCKDH) and subsequent reactions. Acetyl-CoA may be involved in the TCA cycle or other amino acid synthesis. Glutamine can be transported by SLC1A5 (ASCT2), LAT1 (L-type amino acid transporter), and xCT (SLC7A11). Glutamine is involved in glutathione (GSH) synthesis and cell REDOX homeostasis regulation in cytoplasm. In the mitochondria, glutamine produces Glutamate through a reaction catalyzed by glutaminase (GLS), which participates in the TCA cycle by producing α-KG by aminotransferase (ATs) and Glutamate dehydrogenase (GLUD). Created with BioRender.com. (The red blunt line represents inhibition)

BCAAs are a class of fatty side chain amino acids with one branch, including Leu, iLe, and valine. Three BCAAs account for 35% of the essential amino acids in muscle as essential amino acids in the human body. The breakdown process of BCAAs is similar in all species, initially forming branched-chain α-keto acids (BCKAs) via branched-chain amino acid transferase (BCATs) and transferring nitrogen to nitrogen receptors (the most common nitrogen receptor is α-ketoglutaric acid (α-KG) to form glutamate).^45^ The second step is an irreversible rate-limiting reaction catalyzed by branched-chain α-keto acid dehydrogenase (BCKDH), which is phosphorylated and inactivated by the specific kinase BCKDH kinase (BCKDK) and dephosphorylated and activated by Protein phosphatase 1 K (PPM1K). The products are then involved in different physiological activities through further oxidation (Fig. 2).

BCAAs participate in a variety of physiological processes. In terms of metabolism and signaling pathway research, BCAAs, especially Leu, are effective activators of the mTOR signaling pathway. Leu can bind to Sestrin2 (a negative regulator of mTORC1 activity) to promote mTORC1 activation,^46^ thereby promoting protein synthesis in the liver and other tissues.^47^ In addition, BCAAs also promote glycogen absorption by the liver and skeletal muscle and enhance glycogen synthesis.^48^ Furthermore, BCAAs are essential for the proper function of immune cells in the immune system, promoting lymphocyte proliferation and cytotoxic T-cell activation through the oxidative decomposition of dehydrogenase and decarboxylase expressed by immune cells.^49^

### BCAA in cancer

**Fig. 3:**
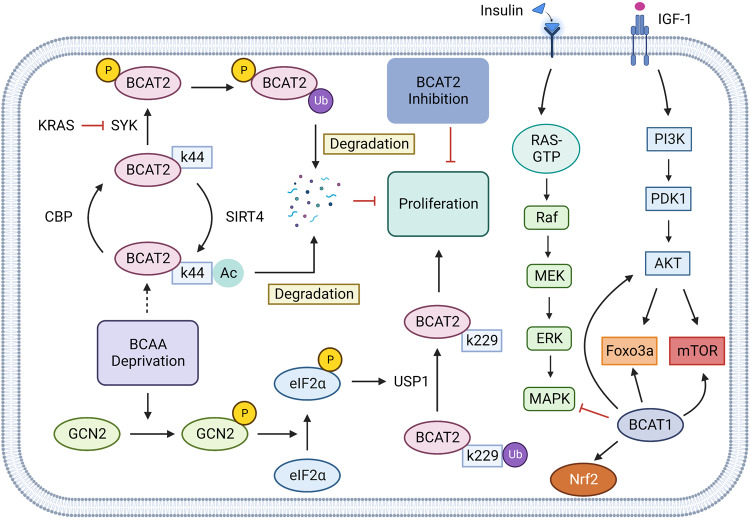
BCAAs metabolism in Cancer. In pancreatic ductal adenocarcinoma (PDAC), KRAS can inhibit the ubiquitination of BCAT2 by spleen tyrosine kinase (SYK) and E3 ubiquitination ligase TRIM21, thereby stabilizing the expression level of BCAT2 in PDAC cells and promoting the proliferation of tumor cells. BCAAs promote Ubiquitin Specific Peptidase 1 (USP 1) through the GCN2-eIF2a pathway and inhibit the degradation of BCAT2 by deubiquitination of the K299 site of BCAT2. This process is inhibited during the BCAAs deprivation. cAMP-responsive Elin-Binding (CREB)-binding protein (CBP) and SIRT4 compete to bind the K44 site of BCAT2, regulating the acetylation level of this site and the degradation of BCAT2. In triple-negative breast cancer, tumor cells can activate MAPK and PI3K/AKT signaling through IGF-1 and insulin signaling, and PI3K/AKT signaling can go on to activate Foxo3a, mTOR signaling, BCAT in the cytoplasm of tumor cells can also promote mitochondrial genesis and mitochondrial function by activating Foxo3a, AKT, mTOR, and Nrf2 to gain survival advantages. Created with BioRender.com. (The red blunt line represents inhibition; The dotted line indicates that the middle step is omitted)

Changes in circulating levels of BCAAs have been reported in cancer patients.^50,51^ Recent metabonomics retrospective studies had shown that increased plasma levels of BCAAs are associated with an increased risk of pancreatic cancer, which was validated in a genetically engineered mouse model of pancreatic ductal adenocarcinoma (PDAC). This phenomenon may be caused by systematic protein breakdown to satisfy the BCAAs needed for its growth during the tumorigenic period.^51^ Moreover, another study suggested that KRAS mutations can promote BCAA metabolism. Although KRAS activation and P53 deletion are present in non-small cell lung cancer (NSCLC) and PDAC, the two tumors utilize BCAA differently despite the same initial events. PDAC cells tend to decompose and utilize extracellular proteins for amino acids, while NSCLC cells extract nitrogen by breaking down circulating BCAAs.^52^ In addition, Lei et al. found that CBP (cAMP-responsive element-binding (CREB)-binding protein) and SIRT4 in PDAC cells bind the K44 site of BCAT2 to acetylate this site, which further promotes the degradation of BCAT2 through the ubiquitin-protein pathway, reduces the metabolic rate of BCAAs in PDAC, and, in turn, inhibits the growth of tumor cells.^53^ In addition, KRAS and USP1 can also regulate the expression of BCAT2 in PDAC through the ubiquitin-proteasome pathway: KRAS can stabilize the expression of BCAT2 in PDAC by inhibiting the ubiquitination of BCAT2 by spleen tyrosine kinase (SYK) and E3 ubiquitination ligase TRIM21,^54^ while USP1 deubiquitinates the K229 site of BCAT2, and BCAAs promote USP1 protein expression at the translation level through the GCN2-eIF2a pathway. Another study found that the expression levels of USP1 and BCAT2 were consistently positively correlated in gene-edited mice and clinical samples.^55^ The Lei’s result further clarified why BCAAs metabolism of PDAC is lower than that of surrounding normal tissues and then turns to other ways to obtain nitrogen (Fig. 3).

**Fig. 4:**
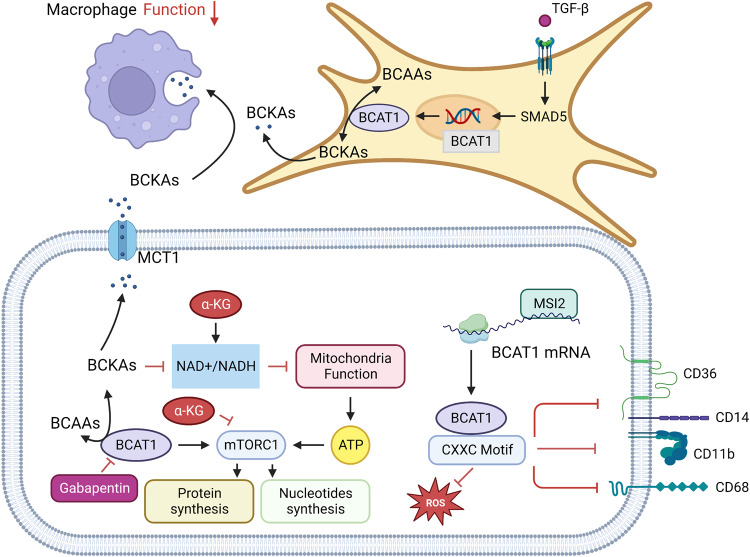
BCAAs metabolism in tumor microenvironment. In triple-negative breast cancer, tumor cells can activate MAPK and PI3K/AKT signaling through IGF-1 and insulin signaling, and PI3K/AKT signaling can go on to activate Foxo3a, mTOR signaling, BCAT in the cytoplasm of tumor cells can also promote mitochondrial genesis and mitochondrial function by activating Foxo3a, AKT, mTOR, and Nrf2 to gain survival advantages. In Leukemia, the RNA-binding protein Musashi 2 (MSI2) binds to BCAT1 mRNA to promote the translation of BCAT1. BCAT1 containing CXXC motif has strong reductive and antioxidant properties, and in wild-type BCAT1 leukemia cells with CXXC motif, The number of cell surface markers CD11b, CD14, CD68, and CD36 decreased. BCKAs excretion in glioblastoma is heavily mediated by monocarboxylate transporter 1 (MCT 1), and the excreted BCKAs are phagocytic and resynthesized into BCAAs by tumor-related macrophages (TAM), but phagocytic activity of macrophages exposed to BCKAs is significantly reduced. BCAT 1 is selectively upregulated in isocitrate dehydrogenase (IDH) wild-type (WT) GBM, alpha-ketoglutaric acid (α-KG) mediates cell death in BCAT 1-deprived IDH WT GBM, and the combination of BCAT 1 inhibitor Gabapentin and α-KG induces tumor cell death.In the tumor microenvironment, CAFs upregulate the transcription of BCAT1 through SMAD5 under the influence of transforming growth factor β (TGF-β) signal, significantly increase the catabolism of BCAAs and secrete BCKAs. PDAC uses BCKAs secreted by CAFs as substrates for BCAAs synthesis or in a BCKDH-dependent mode to promote the increase of BCKA oxidative metabolic flux. Created with BioRender.com. (The red blunt line represents inhibition)

Increasing evidence suggests that elevated plasma BCAAs is a risk factor for pancreatic cancer. Yet, whether elevated circulating BCAAs promotes PDAC progression or PDAC produces more BCAAs. Elevated circulating BCAAs have been observed in both human and mouse models of pancreatic cancer in the early stages of progression, and blood BCAAs levels rise due to excessive protein breakdown in the tissues surrounding pancreatic cancer.^51,56^ Zhu et al. assessed the metabolic reprogramming in tumors and found that metabolic signals were cross-linked between PDAC and CAFs. CAFs significantly increase the catabolism of BCAAs and the secretion of BCKAs in the nutrient-poor tumor microenvironment (TME). PDAC uses BCKAs secreted by CAFs as substrates for BCAAs synthesis or increases the oxidative metabolic flux of BCKA in a BCKDH-dependent mode.^57^ This study suggests the feasibility of targeting BCAAs metabolism in TME mesenchymal and cancer cells for PDAC therapy (Fig. 4).

Lung tumors show higher BCAAs uptake than PDAC. Analysis of labeled BCAAs metabolites showed more labeled α-Ketoisocaproate (α-KIC) and Leu-derived BCKAs in NSCLC cells. Meanwhile BCKDK was highly expressed in NSCLC and regulated ROS production in cells, affecting cell survival.^58^

Interestingly, Chi et al. found that high expression of BCAAs in breast tumor tissues can reduce breast cancer N-cadherin’s expression level and thus inhibit tumor metastasis.^59^ Shafei reported that BCAT1 inhibited the Ras/ERK pathway and activates PI3K/AKT pathway through insulin/IGF-1, ultimately promoting the expression levels of FOXO3a and Nrf2 and regulating the proliferation, migration, and invasion in triple-negative breast cancer (TNBC).^55^ The above studies imply that breast cancers can be classified into subtypes based on their preference for BCAAs metabolism. Another study found that the subtypes of BCATs are correlated with breast cancer subtypes. BCAT1 expressed in the cytoplasm was highly expressed in human epidermal growth factor receptor 2 positive (HER2^+^) breast cancer, while BCAT2 expressed in the mitochondria tended to be highly expressed in estrogen receptor-positive (ER^+^) breast cancer. This suggests that BCATs may regulate tumors through different signaling pathways in different breast cancer subtypes.^60^ Similarly, BCAT1, which is highly expressed in breast cancer cells, promotes mitochondrial production and function by activating the mTOR signaling pathway and ultimately promotes breast cancer cell growth.^61^ The mechanism of action of BCAAs and their metabolic enzymes and metabolites in different breast cancer subtypes still needs further study (Fig. 4).

Silva et al. showed that in glioblastoma (GBM), BCKAs are heavily mediated by monocarboxylate transporter 1 (MCT 1) and that BCKAs expressed in large quantities are phagocytized and resynthesized into BCAAs by tumor-related macrophages (TAM). However, the phagocytic activity of macrophages exposed to BCKAs was significantly reduced.^62^ Overall, BCAAs metabolism has a key role in GBM and that metabolites of BCKAs may have a direct role in tumor immunosuppression. Moreover, recent study found that hypoxia-inducible factor (HIF)−1 and HIF-2 in GBM cells jointly mediate upregulation of the mRNA and protein expression levels of the BCAAs transporter LAT 1 and the BCAAs metabolizing enzyme BCAT1, and ultimately promote the growth of cells under hypoxia conditions.^63^ Furthermore, BCAT 1 is selectively upregulated in isocitrate dehydrogenase (IDH) wild-type (WT) GBM, and α-ketoglutarate (α-KG) mediates cell death in BCAT 1-deficient IDH WT GBM. This argument was supported by the combination of BCAT 1 inhibitor and α-KG induced tumor cell death in patient-derived IDH WT GBM. Mechanistically, high expression of BCAT1 reduces the NAD^+^/NADH ratio, increases mTORC1 activity, and promotes oxidative phosphorylation and nucleotide biosynthesis.^64^ The results of Zhang et al. illustrate the feasibility of targeting BCAAs metabolism in GBM for tumor therapy (Fig. 4).

BCATs are the first enzymes in the BCAAs metabolic pathway, including BCAT c encoded by BCAT 1 gene, mainly expressed in the cytoplasm, and BCAT m encoded by BCAT 2 gene, which is expressed in the mitochondria. BCAT 1 and BCAT 2 share a conserved sequence, the CXXC motif, which has been shown to act as a REDOX switch in BCAT enzymatic action.^65^ However, different isomers react differently to ROS, and the sensitivity of BCAT 2 is many orders of magnitude higher than that of BCAT 1.^66^ On the contrary, BCAT 1 has stronger reducing and antioxidant properties. In acute myeloid leukemia (AML), wild-type (WT) BCAT 1 can metabolize hydrogen peroxide (H2O2), while CXXC motif mutants (CXXS) and wild-type (WT) BCAT 2 cannot. In addition, AML cells overexpressing WT BCAT 1 had lower ROS, and the number of bone marrow markers (CD11b, CD14, CD68, and CD36) that marked cell differentiation on the cell surface was lower, suggesting the involvement of the BCAT 1 CXXC motif in ROS buffering and cell development in AML cells. CXXC motif affects the process of leukemogenesis mediated by ROS. Aberrant activation of BCAT 1 was similarly detected in CML. Hattori et al. revealed that the transcript of BCAT 1 is positively regulated by the oncogenic RNA binding protein Musashi 2 (MSI2), which promotes the production of BCAA in leukemia cells and the development of the disease (Fig. 4).

BCAAs metabolism is altered in various tumors such as PDAC, NSCLC, BRCA, GBM, etc. At present, even in the same type of tumor, different tumor subtypes may have different requirements for BCAAs metabolism and regulatory signaling pathways. In order to achieve precise treatment targeting BCAAs metabolism, we still need to conduct more studies on the relationship and regulatory mechanism between tumor subtypes and BCAAs-related metabolic enzymes and metabolites in the future.

### BCAAs in disease

#### Metabolic disease

Existing studies point out that BCAAs and their metabolites are the strongest biomarkers of metabolic diseases such as obesity, insulin resistance, and type 2 diabetes (T2D).^10^ Elevated BCAAs and their metabolites are key in the early progression of metabolic diseases such as T2D.^60^ Each BCAA has a unique metabolic effect. Yu et al. found that a low-iLe diet can increase liver sensitivity to insulin, increase energy expenditure, and activate the FGF21-UCP1 axis; a low-valine diet has similar but more modest effects as a low-iLe diet, while the low-Leu diet has no effect. Moreover, a low-iLe diet can quickly restore the metabolic health of obese mouse models induced by a high-fat diet.^67^ iLe could act as a regulator of metabolic health and that a low-iLe diet can ameliorate the adverse metabolic effects of obesity. In addition, obesity could inhibit hepatic utilization of BCAAs and cause the inactivation of BCKDH by increasing the ratio of BCKDK (BCKDH kinase)/PPM1K (BCKDH dephosphorylase) in hepatocytes. This phenomenon can be reversed by BCAA diet restriction or regulating the BCKDK/PPM1K ratio in mouse models of obesity and insulin resistance. In addition, White et al. found that the transcription factor ChREBP can also promote BCKDK and inhibit PPM1K expression to inhibit BCKDH activation and promote ATP citrate lyase (ACLY) activation, upregulate the lipid synthesis pathway, and induce hepatic steatosis in the obesity model of high-sugar diet.^68^ Another study showed that knockout of BCAT 2 in white adipose tissue (WAT) confers resistance to high-fat diet-induced obesity through browning of WAT and increased thermogenesis. Mechanistically, acetyl-CoA, a derivative of BCKAs, inhibits the interaction between PR domain-containing protein 16 (PRDM16) and peroxisome proliferator-activated receptor-γ (PPAR-γ) by acetylating the k915 site of PRDM16 to maintain WAT characteristics. When BCAT 2 is knocked down, depletion of BCKAs and its derivative acetyl-CoA promotes WAT brown steatosis and energy expenditure.^69^ In addition, Ma et al. also found that telmisartan, an antihypertensive drug, can directly bind to BCAT2 and inhibit its activity, thereby reducing obesity.

Recently, it was also found that valsartan, an angiotensin II inhibitor, could inhibit BCKDH-BCKDK interaction, decrease BCKDH phosphorylation, and decrease plasma BCAA concentration to increase BCKDH enzyme activity. In addition to valsartan, candesartan and irbesartan have also been found to have similar effects, suggesting that such drugs may have a similar steric structure to bind BCKDK to promote its separation from BCKDH.^70^ BCKDK inhibitors are also effective in attenuating insulin resistance in mouse models of obesity, and the development of a new generation of more powerful BCKDK inhibitors is important for diseases that require inhibition of BCAA catabolism.^71^ In addition, extra-mitochondrial localization of branched-chain α-keto acid dehydrogenase (BCKDH), a rate-limiting enzyme in BCAAs metabolism, has been reported in type 2 diabetic rat model (OLETF). This portion of BCKDH is present on the endoplasmic reticulum (ER) and interacts with AMP deaminase 3 (AMPD3), and is negatively regulated by AMPD3.^72^ Upregulation of AMPD3 has been reported to impair energy metabolism in OLETF hearts. This study further suggested that AMPD3 may induce cardiometabolic changes through AMPD3-BCKDH expression imbalance in cardiomyocytes of diabetic individuals, providing new insights into the mechanism of the development of this disease.

#### Liver and kidney disease

In patients with cirrhosis, enhanced catabolism of BCAAs, increased glutamate synthesis, and decreased circulating BCAAs levels in a hyperammonemia environment have been suggested as hallmarks of the disease and associated with increased risk of hepatic encephalopathy.^73,74^ Elevated circulating BCAAs have been detected in nonalcoholic fatty liver disease (NAFLD). Also, this disturbed BCAAs metabolism has a synergistic effect with the development of T2DM.^75^ Other studies showed that BCAAs supplementation helps restore glucose homeostasis and enhance immune system function in patients with chronic liver disease.^76–78^

In renal disease, circulating BCAAs levels are significantly decreased in patients with chronic renal failure.^79,80^ This phenomenon has been seen in patients with chronic kidney disease (CKD), and a phase II CKD cohort study found that plasma Leu and valine are significantly decreased in CKD patients compared with normal controls.^81^ This may be due to decreased BCAAs levels caused by long-term malnutrition and hemodialysis in CKD patients. Metabolic acidosis also enhances branched-chain amino acid dehydrogenase (BCKD) activity and accelerates protein breakdown. However, supplementing BCAAs and other essential amino acids to patients with chronic renal failure can help maintain protein balance and reduce uremic toxicity.^82–84^

## Aspartate (Asp)

### Aspartate metabolism

**Fig. 5:**
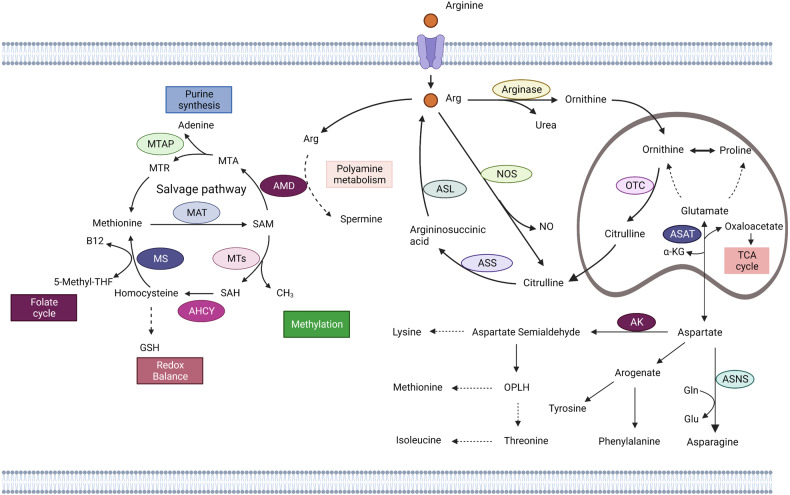
Aspartate, Arginine and Methionine metabolism. Aspartate aminotransferase (ASAT) catalyzes the transfer of amino groups from glutamine to oxaloacetic acid to produce aspartate and α-ketoglutaric acid. Aspartic acid is catalyzed by aspartic synthase (ASNS), and the amino group is provided by glutamine to form asparagine. Aspartic acid can participate in NAD biosynthesis by aspartic oxidase (AO). Aspartate is also involved in the synthesis of Tyrosine and Phenylalanine through its conversion to Arogenate. Aspartic acid can be transformed into Aspartate semialdehyde through aspartic kinase (AK), which further catalyzes o-phospho-l-homoserine (OPLH) to participate in Lysine, Methionine, Threonine, Synthesis of Isoleucine. Arginine in cells is catalyzed by Arginase to produce Ornithine and enter the ornithine cycle. Ornithine transcarbamylase (OTC) catalyzes the production of citrulline in mitochondria. In the cytoplasm, arginine produces citrulline and nitric oxide by nitric oxide synthase (NOS), the first step in the urea cycle. Citrulline is produced by Argininosuccinate synthase (ASS) to arginine, which is catalyzed by Argininosuccinate Lythase (ASL) to produce arginine, and the resultant fumaric acid enters the TCA cycle. In addition, ornithine in mitochondria can be converted from glutamic acid and proline. Methionine can be catalyzed by methionine adenosine transferase (MAT) to produce S-adenosine methionine (SAM). As a methyl donor, SAM participates in the methylation of histones, nucleic acids and proteins under the catalysis of methyltransferase, and produces S-homocysteine (SAH). SAH is catalyzed to produce HOMOcysteine by Adenosylhomocysteinase (AHCY), which may participate in glutathione synthesis (GSH) or in folate recycling and resynthesis of methionine via methionine synthase (MS). In the methionine remedial synthesis pathway, SAM participates in polyamine metabolism via Adenosylmethionine decarboxylase 1 (AMD1), 5,-methylthioadenosine (MTA) is produced and then phosphorylase is re-synthesized through 5-methylthioadenosine (MTAP) and the subsequent reaction. Created with BioRender.com. (The dotted line represents the intermediate process omission)

Asp is an α-amino acid used in protein synthesis that has an α-amino group, an α-carboxylic acid group, and a side-chain carboxamide.^85^ It is a non-essential amino acid because the body can synthesize it. Oxaloacetate is the precursor of Asp. Transaminase transfers amino groups from glutamate to oxaloacetate, producing α-ketoglutarate and Asp. In Asn synthetase-mediated enzymatic reactions, Gln provides an amino group, which combines with β-aspartate-AMP to form asparagine (Asn) and AMP.^86^ Asn is an amino acid necessary for brain development. Since Asp in the blood cannot directly pass through the blood-brain barrier, the development of nerve cells depends on its synthesisation in the brain. When the level of Asn synthetase in the brain is insufficient, the proliferation of brain cells will be limited or even leading to cell death.^13^ In turn, during catabolism, Asn is hydrolyzed by aspartase to Asp, which is then aminated with α-ketoglutarate to form glutamate and oxaloacetic acid. Then oxaloacetic acid enters the citric acid cycle (Fig. 5).^86^

Asp is also a metabolite of the urea cycle, carrying reduction equivalents in the malate-Asp shuttle, providing nitrogen atoms in inosine synthesis, and acting as a hydrogen acceptor in ATP synthesis. Asp is also the precursor of four essential amino acids (methionine, threonine, lysine, and isoleucine). Asp can also act as an amino acid exchange factor, becoming a medium for amino acids in and out of cells, especially histidine, arginine and serine. Asp regulates serine metabolism, nucleotide synthesis, and mTORC1 activity through amino acid exchange factor function.^87^

### Aspartate in cancer

**Fig. 6:**
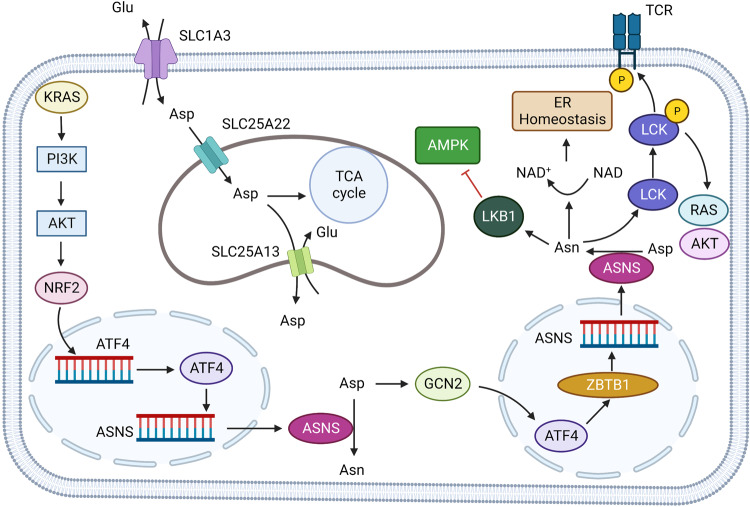
Aspartate metabolism in solid tumor. The high expression of SLC1A3 in tumor cells promoted the absorption of aspartate, supplemented the low aspartate state caused by ASNase, and produced resistance to ASNase therapy. SLC25A22 expressed on mitochondria can increase the intake of mitochondrial aspartate, promote mitochondrial function and reduce oxidative stress. KRAS activates NRF2-ATF4 axis through PI3K/AKT signaling pathway, promotes ASNS transcription and increases intracellular asparagine concentration. Asparagine (Asn) can bind to SRC family tyrosine kinase LCK to assist in phosphorylation of LCK at Tyr394. Enhance LCK activity and T cell receptor signaling, and promote AKT, RAS activation. Asparagine can inhibit AMPK signaling pathway activity by binding to LKB1. In T lymphocytic leukemia cells, ATF4 binds to ASNS gene promoter through ZBTB1 (Zinc Finger and BTB domain-containing protein 1), promotes ASNS transcription, increases intracellular Asparagine concentration. Created with BioRender.com. (The red blunt line represents inhibition)

TP53 is a gene with the highest mutation frequency in human cancer. The protein p53 encoded by this gene inhibits the development of tumors through the regulation of the cell cycle, apoptosis, genomic stability and other pathways.^88–90^ Deng et al. reported that Asp and Asn in colon cancer cell lines could inhibit their activities by binding to LKB1 (encoding filament, threonine kinase, and direct phosphorylation of protein products to activate AMPK), thus inhibiting AMPK-mediated p53 activation.^91^ Activation of p53 can disrupt Asp-Asn homeostasis and promote cell senescence and cycle arrest in lymphoma and colorectal tumor models.^91^ Under hypoxia, Asp is a limiting factor for tumor growth. Hypoxia inhibits the electron transport chain (ETC), affecting energy and Asp synthesis. Garcia-Bermudez et al. studied the sensitivity of tumor cells to mitochondrial ETC inhibitors and found that tumor cells insensitive to ETC inhibition maintain intracellular Asp concentrations through the Asp/glutamate transporter SLC1A3, which gives tumor cells a survival advantage.^92^ In another study on tumor metabolism, Sullivan et al. found that Asp synthesis was a limiting factor for bladder cancer growth when oxygen was lacking in the environment. In bladder cancer cells, the poor permeability of Asp cells prevents the uptake of Asp by tumor cells from the environment. While cells have higher permeability with Asn than Asp, the activity of asparaginase in bladder cancer cells was insufficient, which could not convert Asn into Asp.^93^ After using guinea pig asparaginase 1 (gpASNase1) to promote the conversion of Asn to Asp in tumor cells, the growth rate of tumor cells was significantly increased, suggesting that Asp acquisition is an endogenous metabolic limitation in tumors with difficult Asp acquisition.^93^ It was suggested that Asp is an intrinsic limit to the growth of some tumors in vivo, and breaching this limit will promote tumor growth. The Asp-glutamate transporter SLC1A3 is closely associated with the effect of ETC inhibitors, and the SLC1A3 site is amplified in subclusters of non-glial epithelial tumors and thus against aspartic restriction.^92^ Sun et al. found that SLC1A3 promotes breast cancer cells to L-asparaginase (ASNase) resistance. Also, ASNase consumption of Asp and glutamate could be supplemented by SLC1A3, thus eliminating the inhibitory effect of ASNase and promoting tumor development.^94^ Furthermore, Xu et al. confirmed that overexpressed SLC1A3 in gastric cancer activates the PI3K/AKT pathway, upregulates the expression levels of Glucose transporter 1 (GLUT1), Hexokinase 2 (HK2), and Lactate dehydrogenase A (LDHA), and promotes the growth of gastric cancer, while treatment with the PI3K/AKT inhibitor LY294002 could inhibit the growth-promoting effect of SLC1A3 overexpression on gastric cancer.^95^ Moreover, Wong et al. found that another amino acid transporter, SLC25A22, could promote Asp synthesis, activate the AMPK pathway and reduce oxidative stress in KRAS mutant colorectal cancer (CRC) cells (Fig. 6).^96^ These studies have shown that AATs are potential targets for tumor metabolic reprogramming. Drugs currently being tested that target AATs are shown in (Table 1).

**Table 1:** Drugs that target amino acid metabolism in clinical trials

Name	Research and development code	Target	Disease	Phase	Trial registration number
AXA1125	AXA-1125		NAFLD		NCT04073368
			NASH	Phase 2	NCT04880187
			COVID-19	Phase 2	NCT05152849
BCAT 1 Inhibitor	ERG-24; ERG-245	Basigin (BSG); Branched Chain Amino Acid Transaminase 1 (BCAT1); Matrix Metallopeptidase 2 (MMP2); Matrix Metallopeptidase 9 (MMP9)	cancer/rheumatoid arthritis	Pre-clinical	
BCAT2 modulator		Branched Chain Amino Acid Transaminase 2 (BCAT2)	organic acidemia/diabetes mellitus	Pre-clinical	
BCKDK inhibitor		Branched Chain Keto Acid Dehydrogenase Kinase (BCKDK)	Insulin resistance; Maple glycosuria; Metabolic disorder; Type 2 diabetes	Pre-clinical	
Nanvuranlat	JPH-203	L-type amino acid transporter 1 (LAT1)	Biliary tract carcinoma; Skin allergy; Solid tumor	Phase 2	
4-L-[131I]iodo-phenylalanine	131I-ACD-101	L-type amino acid transporter 1 (LAT2)	glioblastoma	Phase 2	NCT03849105
QBS-10072S	QBS-10072S	L-type amino acid transporter 1 (LAT3)	Advanced solid tumor; Astrocytoma; Cholangiocarcinoma; Glioblastoma; Mesothelioma; Metastatic bladder cancer; Metastatic brain tumor; Metastatic breast cancer; Metastatic colorectal cancer; Metastatic esophageal carcinoma; Metastatic head and neck cancer; Metastatic liver cancer; Metastatic lung cancer; Metastatic ovarian cancer; Metastatic pancreatic cancer; Metastatic prostate cancer; Metastatic renal cell carcinoma; Metastatic gastric cancer; Metastatic urinary tract carcinoma; Sarcoma; Stage IV melanoma; Thymus tumor; Tongue disease; Cervical cancer	Phase 2	NCT04430842
O-(2-[18F] fluoroethyl)-L-tyrosine	TLX101-CDx	L-type amino acid transporter 1 (LAT4)	Glioblastoma; glioma	Clinical	NCT05632562; NCT03451123; NCT03216148; NCT02286531; NCT01579253; NCT01443676
4-L-[124I] iodo-L-phenylalanine	124I-ACD-101	L-type amino acid transporter 1 (LAT5)	Brain tumor	Phase 1	
astatinated IPA	211At-TLX-102	L-type amino acid transporter 1 (LAT6)	Multiple myeloma	Pre-clinical	
R-OKY-034F	OKY-034	L-type amino acid transporter 1 (LAT7)	Pancreatic tumor	Phase 2	
[18F] AA-7	[18 F] NKO-028	L-type amino acid transporter 1 (LAT8)	Cancer; glioma	Clinical	
Crisantaspase	JZP-341	Asparaginase	Acute lymphoblastic leukemia; Adenocarcinoma; Advanced solid tumor; Hematologic tumor; Metastatic colorectal cancer	Pre-clinical	
L-asparaginase	ERY-001	Asparaginase	Metastatic breast cancer; Metastatic pancreatic cancer; Ductal adenocarcinoma of pancreas; Solid tumor	Phase 3	NCT05660473; NCT05631327; NCT05581030; NCT05326984; NCT05326516; NCT04956666; NCT03618238
Pegaspargase biosimilar	PF-690	Asparaginase	Acute lymphoblastic leukemia; hematoma	Pre-clinical	
PJ-017	PJ-017	Asparaginase	Advanced solid tumor	Pre-clinical	
Pegargiminase	ADI-PEG-20	Arginine deiminase (ADI)	Acute myelogenous leukemia; Advanced solid tumor; Glioblastoma; Glioma; Hepatocellular carcinoma; Melanoma; Mesothelioma; Metastatic pancreatic cancer; Non-small cell lung cancer; Prostate tumor; Soft tissue sarcoma; Solid tumor; Uveal melanoma	Phase 3	NCT05616624; NCT05001828; NCT04587830; NCT03449901
Pegzilarginase	AEB-1102	Arginase 1 (ARG1)	Acute myelogenous leukemia; Amino acid and protein metabolism disorders; Melanoma; Myelodysplastic syndrome; Small cell lung cancer; Uveal melanoma	New drug marketing application	
Eryminase		Arginine deiminase (ADI)	Protein metabolism disorder	Pre-clinical	
PFI-102	PFI-102	Peptidyl Arginine Deiminase 4 (PADI4)	Rheumatoid arthritis; Systemic lupus erythematosus	Pre-clinical	
JBI-1044	JBI-1044	Peptidyl Arginine Deiminase 4 (PADI4)	Autoimmune disease; Cancer; Novel coronavirus pneumonia infection (COVID-19); Hidradenitis suppurativa; Inflammatory disease; Metastatic liver cancer; Rheumatoid arthritis; vasculitis	Pre-clinical	
Arginine-depleting enzym	NEI-01		Acute myelogenous leukemia; Advanced solid tumor	Phase 1	NCT05226468
PEGylated arginine degrading enzymes	PJ-016	Arginase (ARG)	Metastatic carcinoma	Drug discovery	
TNG-462	TNG-462	Protein Arginine Methyltransferase 5 (PRMT5)	Advanced solid tumor; Cholangiocarcinoma; Mesothelioma; Metastatic non-small cell lung cancer; Neuro-tumor; Solid tumor	Pre-clinical	
AMG-193	AMG-193	Protein Arginine Methyltransferase 5 (PRMT5)	Advanced solid tumor; Non-small cell lung cancer	Phase 2	NCT05094336
MRTX-9768	MRTX-9768	Protein Arginine Methyltransferase 5 (PRMT5)	cancer	Pre-clinical	
TNG-908	TNG-908	Protein Arginine Methyltransferase 5 (PRMT5)	Advanced solid tumor; Bladder cancer; Cholangiocarcinoma; Glioblastoma; Mesothelioma; Metastatic non-small cell lung cancer; Neuro-tumor; Squamous cell carcinoma	Phase 2	NCT05275478
PRT-543	PRT-543	Protein Arginine Methyltransferase 5 (PRMT5)	Acute myelogenous leukemia; Adenomatoid tumor; Advanced solid tumor; Breast tumor; Chronic granular monocytic leukemia; Diffuse large B-cell lymphoma; Hematologic tumor; Mantle cell lymphoma; Myelodysplastic syndrome; Myelofibrosis; Non-small cell lung cancer; Ovarian tumor; Spina bifida; Uveal melanoma	Phase 1	NCT03886831
PRT-811	PRT-811	Protein Arginine Methyltransferase 5 (PRMT5)	Advanced solid tumor; Brain tumor; Glioblastoma; Glioma; myelofibrosis	Phase 1	NCT04089449
MRTX-1719	MRTX-1719	Protein Arginine Methyltransferase 5 (PRMT5)	Advanced solid tumor	Phase 2	NCT05245500
Onametostat	JNJ-64619178	Protein Arginine Methyltransferase 5 (PRMT5)	Advanced solid tumor; Myelodysplastic syndrome; Non-hodgkin’s lymphoma	Phase 1	NCT03573310
PRMT-5 inhibitors	CTx-0262135	Protein Arginine Methyltransferase 5 (PRMT5)	Cancer; hematopathy	Drug discovery	
SKL-27969	SKL-27969	Protein Arginine Methyltransferase 5 (PRMT5)	Advanced solid tumor	Phase 2	NCT05388435
SYHX-2001	SYHX-2001	Protein Arginine Methyltransferase 5 (PRMT5)	Acute myelogenous leukemia; Adenomatoid tumor; Advanced solid tumor; Hematologic tumor; Melanoma; Pancreatic tumor	Phase 1	NCT05407909
GSK-3226593	GSK-3226593	Protein Arginine Methyltransferase 5 (PRMT5)	Soft tissue sarcoma	Pre-clinical	
SH-3765	SH-3765	Protein Arginine Methyltransferase 5 (PRMT5)	Advanced solid tumor; Non-hodgkin’s lymphoma	Phase 1	NCT05015309
AM-9747	AM-9747	Protein Arginine Methyltransferase 5 (PRMT5)	Cancer	Pre-clinical	
PF-06939999	PF-06939999	Protein Arginine Methyltransferase 5 (PRMT5)	Advanced solid tumor; Endometrial carcinoma; Metastatic bladder cancer; Metastatic esophageal carcinoma; Metastatic head and neck cancer; Metastatic non-small cell lung cancer; Squamous cell carcinoma; Cervical cancer	Phase 1	NCT03854227
AGX-323	AGX-323	Protein Arginine Methyltransferase 5 (PRMT5)	Cancer	Pre-clinical	
ALG-070043	ALG-070043	Protein Arginine Methyltransferase 5 (PRMT5)	Hepatocellular carcinoma; Non-small cell lung cancer	Pre-clinical	
PRT-220	PRT-220	Protein Arginine Methyltransferase 5 (PRMT5)	Graft versus host disease	Pre-clinical	
OATD-02	OATD-02	Arginase 1 (ARG1); Arginase 2 (ARG2)	Advanced solid tumor; Metastatic colorectal cancer; Metastatic ovarian cancer; Metastatic pancreatic cancer; Metastatic renal cell carcinoma	Phase 1	NCT05759923
IO-112	IO-112	Arginase 1 (ARG1)	Solid tumor	Phase 1	NCT03689192
Resminostat	YHI-1001	Arginase 1 (ARG1); Histone deacetylase (HDAC); Histone Deacetylase 1 (HDAC1); Histone Deacetylase 2 (HDAC2); Histone Deacetylase 3 (HDAC3); Histone Deacetylase 6 (HDAC6)	Biliary tract tumor; Cholangiocarcinoma; Colorectal cancer; Cutaneous T-lymphoblastoma; Gallbladder tumor; Hepatocellular carcinoma; Hodgkin,s lymphoma; Mycosis fungoides; Non-small cell lung cancer; Pancreatic neoplasm; Sezary syndrome; Solid tumor	Phase 2	NCT02400788
CB-280	CB-280	Arginase (ARG)	Cystic fibrosis	Phase 1	NCT04279769
Arginase inhibitor	AZD-0011	Arginase (ARG)	Cancer	Pre-clinical	
Numidargistat	INCB-01158	Arginase (ARG); T cell receptor gene (TCR)	Advanced solid tumor; Biliary tract carcinoma; Bladder cancer; Cancer; Colorectal cancer; Endometrial carcinoma; Esophageal tumor; Head and neck tumors; Lung tumor; Melanoma; Mesothelioma; Multiple myeloma; Non-small cell lung cancer; Ovarian tumor; Renal cell carcinoma; Squamous cell carcinoma; Gastric tumor; Transitional cell carcinoma	Phase 2	NCT03314935; NCT02903914
OATD-05	OATD-05	Arginase (ARG)	Cancer	Drug discovery	
Pegylated, cobalt-replaced human arginase	PT-01	Arginase (ARG)	Cancer; Metastatic carcinoma; Metastatic liver cancer; Stage III melanoma; Stage IV melanoma	Phase 1	NCT04136834
Bicyclic arginase inhibitors		Arginase 1 (ARG1)	Cancer	Pre-clinical	
C-0021158	C-0021158	Arginase 2 (ARG2)	Cancer	Drug discovery	
Pegylated human arginase	PEG-BCT-100	Arginase (ARG)	Acute lymphoblastic leukemia; Acute myelogenous leukemia; Advanced solid tumor; Cancer; Glioma; Hepatocellular carcinoma; Hormone-resistant prostate cancer; Neuroblastoma; Renal cell carcinoma; Retinopathy; Sarcoma; Stage IV melanoma	Phase 2	NCT03455140; NCT02899286; NCT02285101; NCT02089633; NCT02089763; NCT01092091; NCT00988195
AB-474	AB-474	Arginase 1 (ARG1)	Cancer	Pre-clinical	
ZB-49-0010	ZB-49-0010	Arginase 2 (ARG2)	Atherosclerosis; Cardiovascular diseases; hypertension	Pre-clinical	
SCR-6920	SCR-6920	Methylthioadenosine Phosphorylase (MTAP); protein arginine N-methyltransferase (PRMT); Protein Arginine Methyltransferase 5 (PRMT5)	Advanced solid tumor; Hematologic tumor; Metastatic non-small cell lung cancer; Non-hodgkin,s lymphoma	Phase 1	NCT05528055
Telaglenastat hydrochloride	CB-839	Glutaminase (GLS)	Cervical cancer	Pre-clinical	
Sirpiglenastat	DRP-104	Glutaminase (GLS)	Advanced solid tumor; Autoimmune disease; Cancer; AIDS related dementia syndrome; Inflammatory disease; Laryngeal tumor; Lung tumor; Metastatic non-small cell lung cancer; Oral tumor; Throat tumor; Squamous cell carcinoma; Urinary tract tumor	Phase 2	NCT04471415
IPN-60090	IPN-60090	Glutaminase (GLS)	Ovarian tumor	Pre-clinical	
Macrocyclic glutaminase 1		Glutaminase (GLS)	Advanced solid tumor	Pre-clinical	
BPTES	D-JHU-29	Glutaminase (GLS)	Breast tumor; Hematologic tumor; Pancreatic neoplasm; Rett syndrome	Pre-clinical	
RP-10107	RP-10107	Glutaminase (GLS)	Solid tumor	Pre-clinical	
DRP-367	DRP-367	Glutaminase (GLS)	Autoimmune disease; Cancer; Inflammatory disease	Drug discovery	
Tiptuximab		Glutaminase (GLS)	Cancer; Non-small cell lung cancer	Pre-clinical	
Kidney mitochondrial glutaminase inhibitors		Glutaminase (GLS)	Cancer	Pre-clinical	
Sirpiglenastat	JHU-083	Glutaminase (GLS)	Advanced solid tumor; Autoimmune disease; Cancer; AIDS related dementia syndrome; Inflammatory disease; Laryngeal tumor; Lung tumor; Metastatic non-small cell lung cancer; Oral tumor; Throat tumor; Squamous cell carcinoma; Urinary tract tumor	Phase 2	
xCT inhibitor		Solute Carrier Family 7 Member 11 (SLC7A11)	Ovarian tumor	Pre-clinical	
Florilglutamic acid (18F)	BAY-94–9392	Solute Carrier Family 7 Member 11 (SLC7A11)	Cancer; Hepatocellular carcinoma	Phase 1	
DC-10	DC-10	Solute Carrier Family 7 Member 11 (SLC7A11)	Cancer	Pre-clinical	
AX-09	AX-09	Solute Carrier Family 7 Member 11 (SLC7A11)	Colorectal cancer; Metastatic breast cancer; Non-small cell lung cancer	Pre-clinical	
xCT-mAb	AbX-09	Solute Carrier Family 7 Member 11 (SLC7A11)	Colorectal cancer; Metastatic breast cancer; Non-small cell lung cancer	Pre-clinical	
Cysteine/cystine	PR0–071	Solute Carrier Family 7 Member 11 (SLC7A11)	Central nervous system diseases; Impulse control disorder; Obsessive compulsive disorder; Schizophrenia; trichotillomania	Phase 2	
MEDI-7247	MEDI-7247	Solute Carrier Family 1 Member 5 (SLC1A5)	Hematologic malignancy	Phase 1	NCT03811652; NCT03106428
IDE-397	IDE-397	Methionine Adenosyltransferase 2A (MAT2A)	Advanced solid tumor; Metastatic bladder cancer; Metastatic esophageal carcinoma; Metastatic head and neck cancer; Metastatic non-small cell lung cancer; Metastatic pancreatic cancer; Metastatic gastric cancer	Phase 2	NCT04794699
S-95035	S-95035	Methionine Adenosyltransferase 2A (MAT2A)	Solid tumor	Pre-clinical	
AG-270	AG-270	Methionine Adenosyltransferase 2A (MAT2A)	Advanced solid tumor; Lymphoma; Metastatic non-small cell lung cancer; Ductal adenocarcinoma of pancreas	Phase 1	NCT03435250
SCR-7952	SCR-7952	Methionine Adenosyltransferase 2A (MAT2A)	Cancer; Solid tumor	Pre-clinical	
S-095033	S-095033	Methionine Adenosyltransferase 2A (MAT2A)	Metastatic esophageal carcinoma; Squamous cell carcinoma	Pre-clinical	
Evexomostat	SDX-7320	Methionyl Aminopeptidase 2 (METAP2)	Cancer; Hepatocellular carcinoma; Idiopathic pulmonary fibrosis; Metastatic breast cancer; Metastatic colorectal cancer; Metastatic non-small cell lung cancer; Metastatic prostate cancer; Type 2 diabetes mellitus; Prostatic tumor	Phase 2	NCT05570253; NCT02743637
APL-1202	APL-1202	Methionyl Aminopeptidase 2 (METAP2)	Bladder cancer	Phase 3	NCT04813107; NCT04736394; NCT04601766; NCT04498702; NCT04490993; NCT03672240
M-8891	M-8891	Methionyl Aminopeptidase 2 (METAP2)	Advanced solid tumor; cancer	Phase 1	
SDX-7195	SDX-7195	Methionyl Aminopeptidase 2 (METAP2)	Metabolic disorder	Pre-clinical	NCT04073368

Asparagine has received extensive attention as a new target for cancer treatment. Knott et al. reported that the expression level of asparagine synthetase (ASNS) in breast cancer is closely related to metastatic recurrence and that inhibition of ASNS or restriction of dietary Asn can reduce tumor metastasis.^97^ In non-small cell lung cancer (NSCLC), activating transcription factor 4 (ATF4) can alter amino acid uptake and increase Asn synthesis through AKT and NRF2 downstream of KRAS. In addition, the use of AKT inhibitors in combination with extracellular asparagine (ASN) depletion can significantly inhibit tumor growth (Fig. 6).^98^

Asn also has a key role in the growth and function of immune cells. Hope et al. found that CD8^+^T cells hardly express asparagine synthase (ASNS) during the early stage of CD8^+^T cell activation and that the growth, activation, and metabolic reprogramming of CD8^+^T cells are disrupted in the context of Asn deprivation.^99^ Wu et al. also demonstrated that Asn levels are increased in activated CD8^+^T cells and bind to the SRC family tyrosine kinase LCK, assisting in the phosphorylation of LCK at Tyr394 and 505, enhancing LCK activity and T-cell receptor signaling.^100^ Asn also has a key role in hematological malignancies. Williams et al. found that activating transcription factor 4 (ATF4) binds to the ASNS gene promoter through Zinc Finger and BTB domain-containing protein 1 (ZBTB1) to promote ASNS transcription in drug-resistant T-cell leukemia. However, ZBTB1 null T-cell leukemia cells are sensitive to ASNase (Fig. 6).^101^ The current use of bacterial-derived L-asparaginase (ASNase) in pediatric acute lymphoblastic leukemia (ALL) has significantly improved cure rates.^102^ However, in solid tumors, several clinical trials have shown the occurrence of drug-related toxic side effects such as pancreatitis, neutropenia, and hypoproteinemia.^103–105^ These toxic side effects are caused, at least in part, by the synergistic activity of glutaminase in ASNase.^106,107^ Based on the purpose of improving the efficacy of ASNase in hematological malignancies, expanding the use of ASNase and reducing side effects, a new generation of ASNase is being developed (Tables 1, 2).

**Table 2:** Approved drugs targeting amino acid metabolism

Name	Research and development code	Target	Disease	Status
Sodium phenylbutyrate	ACER-001	Branched Chain Keto Acid Dehydrogenase Kinase (BCKDK)	Maple glycosuria; Disturbance of urea cycle	Approved
Pegaspargase	NSC-109229	Asparaginase	Acute lymphoblastic leukemia; Acute B lymphoblastic leukemia	Approved
Crisantaspase	OP-01	Asparaginase	Acute lymphoblastic leukemia	Approved
Calaspargase pegol	EZN-2285	Asparaginase	Acute lymphoblastic leukemia; Ductal adenocarcinoma of pancreas	Approved
Crisantaspase	JZP-458	Asparaginase	Acute lymphoblastic leukemia; Lymphocytic leukemia	Approved
Asparaginase		Asparaginase	Acute lymphoblastic leukemia	Approved
Arginine pidolate	G-278		Cognitive impairment	Approved

### Aspartate in disease

#### Immune disease

Abnormal metabolism of immune cells in autoimmune diseases can promote the chemotaxis of inflammatory cells and the production of inflammatory factors. In rheumatoid arthritis (RA), overproduction of the cytokine tumor necrosis factor (TNF) is a central event in pathogenesis, and endoplasmic reticulum (ER) rich T cells are the major releasers of TNF in inflamed joints.^108,109^ Wu et al. found that the abundance of mitochondrial Asp in T cells in rheumatoid arthritis (RA) was decreased, which inhibited NAD^+^ turnover, resulting in a decrease in NAD^+^/NADH ratio and a reduction in ADP-ribosylation of proteins which is NAD^+^ dependent. The absence of ADP ribosylation of the endoplasmic reticulum (ER) chaperone BiP releases ER stress proteins, driving ER dilation and TNF production. Moreover, treating T cells in rheumatoid arthritis with exogenous NAD^+^ or Asp prevents ER expansion and suppresses RA inflammation.^12^

The treatment strategy for RA and other autoimmune diseases is to use antibodies to block cytokines or their receptors. The latest small molecule inhibitors are targeted Janus kinase (JAK) inhibitors.^110^ These therapeutic strategies are designed to block the downstream practice of inflammatory pathways. However, these downstream signaling pathways are widely distributed in cell types other than immune cells, which contributes to adverse events such as thrombosis.^111,112^ Therefore, the research on upstream inflammation in autoimmune diseases is helpful in preventing the development of the disease from the source.

#### Neurological disease

Asparagine synthesis disorder (ASD) is a newly discovered neurological disorder associated with mutations in the ASNS gene on chromosome 7q2. ASD seriously impacts early neurodevelopment, leading to intellectual disability, developmental delay, intractable seizures, progressive brain atrophy and respiratory defects.^13–18^ Currently, the disease can only be diagnosed by DNA sequencing, and only a subset of individuals have detectable reductions in Asn levels in serum and cerebrospinal fluid, hindering the use of this test for initial screening. Because Asn does not actively accumulate in the brain due to the blood-brain barrier, reduced activity of ASNS in the brain is thought to contribute to the disease.^13,14^ So far, 15 ASD-related mutations have been reported, some of which disrupt the protein structure and reduce the substrate binding ability and catalytic efficiency of ASNS. For example, R49Q is a mutation located in the Gln-binding pocket of the N-terminal domain, and this mutation causes the loss of hydrogen bonds not only to the second β-sheet but also to Gln. Moreover, G289A and T337I mutations are located proximal to the ATP-binding pocket of the C-terminal domain, G289A would cause steric conflict with Ser293, and T337I would cause a hydrophobic patch on the protein surface and reduce protein solubility.^16^

In terms of treatment, dietary Asp supplementation has not been as effective as expected, and artificially elevated blood Asn may affect the absorption of other amino acids due to competition for cotransporters.^113,114^ Current treatments are only partially effective, and further understanding of the disease’s mechanism are needed to develop effective drugs.

In neurological diseases, functional defects in N-methyl-D-aspartate receptors (NMDARs) are the major defects that cause neural signaling disorders. NMDARs are a class of glutamate and ion channel receptors. NMDA receptor signaling is mediated by Ca2^+^ permeability and the C-terminal domain of GluN2 subunit-associated network signaling and scaffold proteins. Mutations in NDMARs subunits are associated with neurodevelopmental disorders.^115,116^ D-aspartate (D-Asp) has been shown to influence the signaling of NMDARs by acting as an agonist to bind to the agonist site of NMDARs and activate this glutamate receptor. D-Asp is present in the cytoplasm, peroxisome and extracellular neurons. Endogenous D-Asp is converted from L-Asp by racemization in the central nervous system and endocrine system.^117^ Several preclinical studies have shown that D-Asp is associated with the NMDA-dependent phenotype associated with schizophrenia (Sch). In a D-Asp oxidase knockout mouse model, treatment with D-Asp significantly alleviated phencyclidine-induced cortico-limbic thalamic dysfunction and reduced neuronal prepulse deficits induced by psychotropic drugs (amphetamine and MK-801).^118,119^ Sacchi et al. found that extracellular D-Asp and L-Glu levels were increased in the prefrontal cortex of olanzapine-treated mice but not in a D-Asp oxidase knockout mouse model. Regulation of D-Asp metabolism in the central nervous system may have an impact on olanzapine treatment in patients with drug-resistant schizophrenia (TRS).^120^ Currently, research on D-Asp as a treatment for TRS disease is still in its early stages, and animal experiments are ongoing.

## Glutamine (Gln)

### Glutamine metabolism

Gln is an α-amino acid used in protein synthesis. It is structurally similar to glutamate, but the carboxylic acid group of the side chain is replaced by an amide. Gln is a non-essential amino acid obtained from food^121^ and the most consumed amino acid and is involved in synthesizing all nonessential amino acids (NEAAs) and proteins.^9^ Muscle tissue produces the most Gln in the human body, accounting for about 90% of all synthesized Gln. The brain and lungs can also release a small amount of Gln. Although the liver can also synthesize Gln, its main function is to regulate the large amount of Gln absorbed from the intestine. Gut cells, kidney cells, activated immune cells, and various tumor cells are the most urgent consumers of Gln.^122–124^ Gln enters the cell via the amino acid transporter ASCT2/SLC1A5 and is converted to glutamate in the mitochondria by a deamination reaction involving glutaminase (GLS). Glutamate is then catalyzed by glutamate dehydrogenase (GDH) or glutamate transaminase, or aspartate transaminase (TAs) to produce α-ketoglutarate (α-KG). α-KG is an intermediate product of the TCA cycle (Fig. 5). Under hypoxia or mitochondrial dysfunction, α-KG can be converted to citrate by Isocitrate dehydrogenase (IDH 2) catalyzed carboxylation reaction, which is used for the synthesis of amino acids and fatty acids and the production of reducing agent NADPH.^125–127^

### Glutamine in cancer

**Fig. 7:**
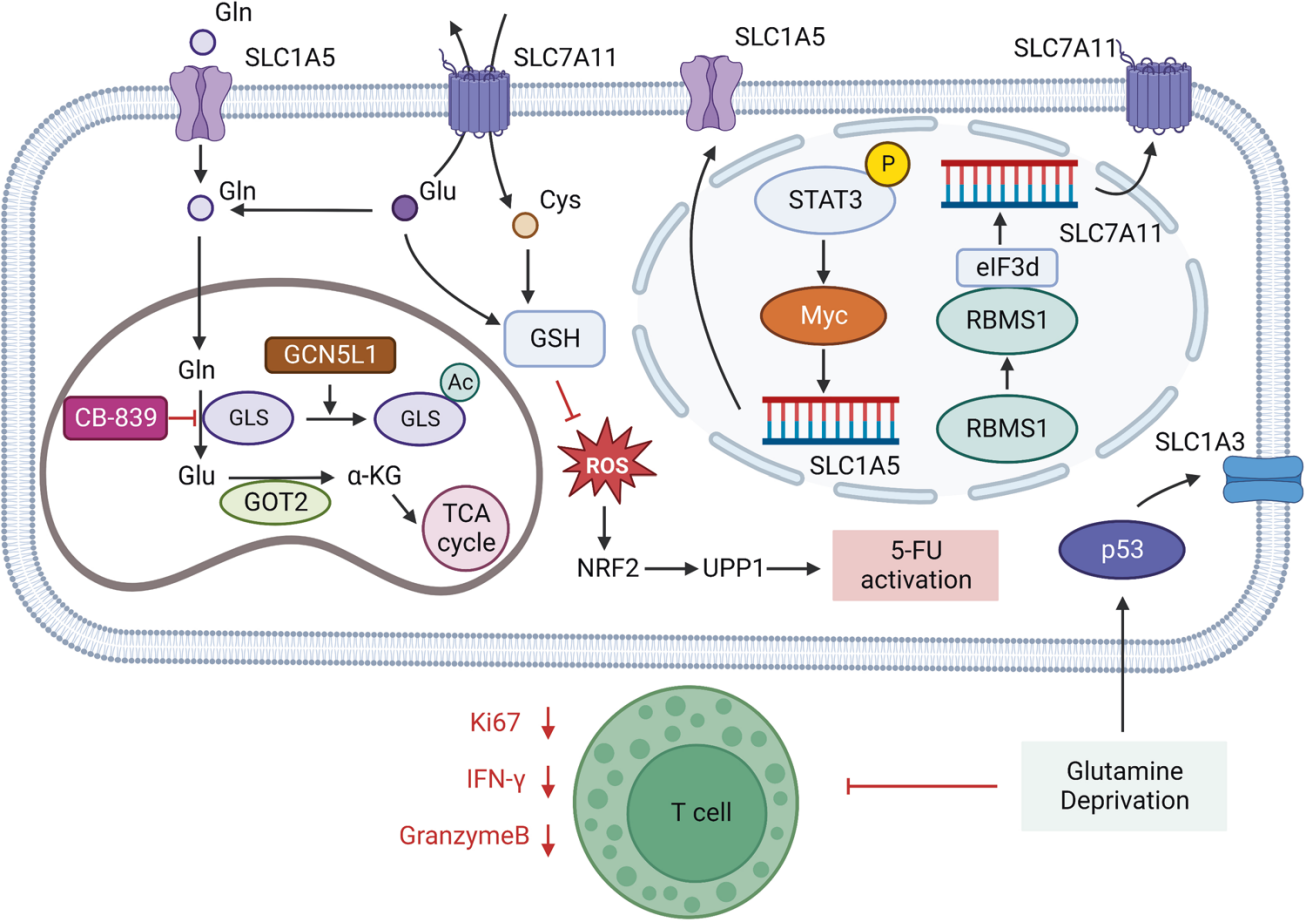
Glutamine metabolism in tumor. GCN5L1 (general control of amino acid synthesis 5 like 1) in mitochondria can promote the acetylation and inactivation of GLS, thus inhibiting the activation of mTORC1 and cell proliferation. GOT2 catalyzes the production of α-KG (α-ketoglutaric acid) from glutamate. When the expression level of GOT2 is decreased, the participation of Glu in the synthesis of GSH increases and Glu is sensitive to the glutaminase inhibitor CB-839. Treatment with CB-839 increased ROS (reactive oxygen species) levels and promoted the activation of 5-FU through the NRF2 (Nuclear factor erythroid 2-related factor 2)-UPP1 (Uridine phosphorylase 1) axis. SASP(Sulfasalazine) reduces intracellular glutamate and extracellular cystine exchange by inhibiting SLC7A11. Glutamine deprivation increases the expression of SLC1A3 on the surface of colon cancer cells by stimulating p53. In glutamine depletion environment, T cells secreted less Granzyme B and IFN-γ, and their function was inhibited. Acute myeloid leukemia (AML) cells promote SLC1A5 transcription via the STAT3-MYC axis. RNA-binding protein RBMS1 in lung cancer promotes SLC7A11 translation by binding to eIF3d. Created with BioRender.com. (The red blunt line represents inhibition)

Tumor cells are urgent consumers of Gln. The signaling molecules Akt, Ras, and AMPK can induce lactate production by activating glycolysis to cause the Warburg effect, prompting tumor cells to meet energy demand through Gln metabolism. Gln metabolism is regulated by oncogenes/tumor suppressor genes such as c-Myc and p53 in various tumors.^128^ The oncogene c-Myc upregulates Gln metabolism through transcriptional activation of GLS and SLC1A5 genes. Mukha et al. reported that GLS-driven Gln metabolism is a regulator of radiotherapy tolerance in prostate cancer (PCa) and that high expression of GLS 1 and c-MYC, key regulators of Gln, are significantly associated with reduced progression-free survival in prostate cancer patients treated with radiotherapy. Gln metabolism can maintain prostate cancer stem cells (CSCs) through α-KG-dependent chromatin dioxygenase. Inhibition of Gln metabolism reduces the frequency of CSCs population in vivo and the rate of tumor growth in mouse models.^129^ Amaya et al. found that signal transducers and activators of transcription 3 (STAT3) promote MYC expression in tumor cells in AML, which in turn regulates the transcription of amino acid transporter SLC1A5, promotes Gln metabolism in AML cells, and oxidative phosphorylation (OXPHOS) of leukemia stem cells (LSCs). Small-molecule inhibitors of STAT3 selectively kill AML stem cells and preserve normal hematopoietic cells.^130^ In addition, Tajan et al. found that in colon cancer, Gln deprivation stimulates p53 activation and promotes the expression of the aspartate/glutamate transporter SLC1A3, thereby promoting glutamate, Gln and nucleotide synthesis, maintaining electron transport chain and tricarboxylic acid cycle activity. Loss of SLC1A3 reduces tumor cell resistance to Gln starvation and inhibits tumor cell growth.^131^ In addition, it has been shown that tumor cells with high expression of cystine/glutamate anti-transporter SLC7A11/xCT are highly dependent on Gln metabolism. In the absence of amino acids such as cystine, cells promote translation of ATF4 via the general control non-repressor 2 (GCN2) -eukaryotic initiation factor (eIF2a) signaling pathway, which promotes transcription of genes involved in amino acid metabolism and stress response, including SLC7A11, enabling cells to cope with amino acid starvation.^132^ In lung cancer, the RNA-binding protein RBMS1 was reported to interact directly with eIF3d to promote SLC7A11 translation (Fig. 7).^133^ Because tumor cells exchange intracellular glutamate for extracellular cystine through SLC7A11, intracellular glutamate is consumed, which causes cells to absorb more Gln and activate glutaminase to supplement intracellular glutamate, making cells with high expression of SLC7A11 become Gln-dependent. In triple-negative breast cancer (TNBC), cells with high SLC7A11 expression consume more Gln and are more sensitive to Gln starvation compared to other breast cancer cells.^134^ SLC7A11 is also highly expressed in lung, PDAC, renal, and liver cancers.^135–137^ Moreover, Badgley et al. found that deletion of SLC7A11 has no effect on normal pancreatic tissue development in mice but severely impairs KRAS-driven PDAC growth.^137^ The non-necessity of SLC7A11 under physiological conditions and the high expression of SLC7A11 in tumors make SLC7A11 a promising target for cancer therapy (Table 1).

Glutaminase, which hydrolyzes Gln to glutamate, is a key enzyme in Gln metabolism. The expression of glutaminase is tissue-specific. Glutaminase is actively expressed in periportal liver cells, renal epithelial cells, and the central nervous system, which is used to synthesize urea and neurotransmitters. Four isoforms of human glutaminase are divided into two highly active renal glutaminase types encoded by GLS1 and two low active hepatic glutaminase types encoded by GLS2.^138^ The heterogeneity of GLS1 and GLS2 expression in different tumors indicates that malignant cells have different requirements for Gln metabolism. Zhang et al. found that general control of amino acid synthesis 5 like 1 (GCN5L1) in mitochondria of liver cancer cells can promote the acetylation and inactivation of GLS1 and GLS2 isomers, thus inhibiting mTORC1 activation and cell proliferation. In a mouse model of hepatocellular carcinoma induced by diethylnitrosamine (DEN), liver GCN5L1 knockout increased DEN sensitivity in the model. In addition, hepatoma cells with low expression of glutamic oxalacetic transaminase 2 (GOT2) showed sensitivity to the glutaminase inhibitor CB-839. Specifically, hepatoma cells with low expression of GOT2 showed a high dependence on Gln metabolism by increasing Gln metabolism, nucleotide synthesis, and glutathione synthesis to support cellular antioxidants (Fig. 7).^139^ Interestingly, in prostate cancer treated with androgen deprivation therapy, Xu et al. found that although androgen deprivation therapy inhibited the expression of renal glutaminase (KGA) in the GLS1 subtype, the expression of glutaminase C (GAC) was upregulated in tumor cells, which is an androgen-independent GLS1 subtype with stronger enzymatic activity. This switch leads to increased Gln utilization by tumor cells and promotes tumor proliferation and metastasis. Therapeutic approaches inhibiting GAC may increase the efficacy of castration-resistant prostate cancer.^140^ In clear-cell ovarian cancer (OCCC), the glutaminase inhibitor CB-839 inhibited ARID1A (AT-rich interactive domain-containing protein 1A) -mutated PDX tumor growth. ARID1A is a member of the SWI/SNF family, and the inhibition of GLS1 by SWI/SNF is weakened in OCCC with ARID1A mutation, which promotes Gln utilization and metabolism IN tumor cells.^141^ SWI/SNF mutations are present in nearly 25% of cancers, which led us to wonder whether other SWI/ SNF-mutated tumors are also sensitive to glutaminase inhibitors.^142,143^

Best et al. reported that LKB1 mutation in KRAS mutant lung adenocarcinoma confers a glutamate enriched phenotype in TME, and this feature was associated with CD8^+^T cell activation against PD-1, whereas treatment with the glutaminase inhibitor CB-839 inhibited CD8^+^T cell expansion and activation. Their data suggested that glutaminase inhibitors could inhibit CD8^+^T cells activated by PD-1 immunotherapy in lung adenocarcinoma.^144^ Morevoer, Zhao et al. reported that CB-839 could promote the production of reactive oxygen species (ROS) in colorectal cancer cells, cause nuclear translocation of Nrf2, and subsequently upregulate the expression of uridine phosphorylase 1 (UPP1), which promotes the activation of 5-fluorouracil (5-FU).^145^ Existing preclinical and clinical experiment data show that Gln inhibitor CB-839 joint capecitabine can effectively treat type PI3KCA mutations in colorectal cancer (NCT02861300).

A randomized, double-blind, controlled phase II trial in advanced renal cell carcinoma (RCC) demonstrated synergistic anticancer effects with the combination of the glutaminase inhibitor telaglenastat (CB-839) and the mTOR inhibitor everolimus (TelaE), which was well tolerated by patients previously treated with TKIs. Also, TelaE could improve progression-free survival (PFS) compared with placebo plus everolimus (PboE).^146^ Another phase I b clinical trial also showed good tolerability and clinical activity of TelaE or telaglenastat combined with cabozantinib (TelaC) in treating RCC.^147^

### Glutamine in disease

#### Pancreatitis

Gln can be used as a nutritional supplement for a variety of diseases. Several meta-analyses found that Gln supplementation can reduce the mortality, complication rate, and total length of hospital stay for patients with severe pancreatitis.^148–151^ A randomized, double-blind, placebo-controlled clinical study showed that supplementation of Gln to the low fermentable oligo-monosaccharides and polyols (FODMAP) diet improves irritable bowel syndrome (IBS) symptoms.^152^ In terms of promoting wound healing, Arribas-Lopez et al. found that arginine and Gln supplementation could positively affect wound healing, and Gln supplementation significantly affected nitrogen balance in patients and reduced the length of hospital stay and mortality.^153^ However, Gln supplementation does not seem to significantly affect the prognosis of burn patients. Moreover, in a double-blind, randomized, placebo-controlled trial enrolling 1200 patients, survival to hospital discharge was 40 days in the Gln-supplementation group versus 38 days in the placebo group. Mortality was 17.2% in the Gln group, which was not significantly different from 16.2% in the placebo group, and Gln supplementation did not reduce the length of hospital stay.^154^ In their study, Heyland et al. showed the benefits and risks of Gln supplementation, while clinical trials in burns and other diseases have shown conflicting results. The benefits and risks of Gln supplementation in various diseases still need more data from clinical trials.

#### Cardiovascular disease

In cardiovascular disease, Myc and Myc-related factor X (Max) upregulate the Gln transporters SLC1A5 and SLC7A5 and mitochondrial malate in pulmonary hypertension, thereby promoting glutaminolysis-induced right ventricular hypertrophy.^19^ Under oxidative stress, the glutathione (GSH) level in cardiomyocytes decreases by 60–70%, and the levels of Gln, glutamate and α-ketoglutarate (α-KG) also decrease significantly, while the enzyme activity of GLS, which converts Gln to glutamate, is enhanced. However, inhibition of GLS activity can reduce ATP and GSH levels produced by cardiomyocytes under oxidative stress conditions.

T2DM is a major risk factor for the development of cardiovascular disease. Dysregulation of skeletal muscle metabolism in diabetes affects insulin sensitivity and glucose homeostasis. Dollet et al. found that Gln is a key amino acid in the regulation of glucose stability and insulin sensitivity, and the level of Gln affects the inflammatory response of skeletal muscle and regulates the expression of the adaptive protein GRB10, an insulin signaling inhibitor. Moreover, the systemic elevation of Gln improves insulin sensitivity and restores glucose homeostasis in mouse models of obesity.^155^

The anthracycline antibiotic doxorubicin (DOX) is a widely used anti-tumor drug in solid malignant tumors; yet, this therapy may lead to serious cardiotoxicity due to free radicals and oxidative stress. Gln supplementation significantly reduced cardiac lipid peroxide levels and increased peroxidase and glutathione levels, protecting cardiac function in DOX-treated rat models.^156,157^

Drugs targeting cardiac Gln metabolism are being developed. Oridinon (Ori), a natural terpenoid derived from the plant Isodon rubescens (Hemsl.), can increase cardiac Gln levels and inhibit the decline of ATP/ADP ratio, protecting cardiomyocytes and reducing infarct size in a rat model of myocardial injury.^158^

#### Severe acute respiratory syndrome

Severe acute respiratory syndrome coronavirus 2 (SARS-CoV-2) is the cause of coronavirus disease 2019 (COVID-19). The disease is spread through close person-to-person contact or respiratory secretions from infected people. Risk factors for COVID-19 include cardiovascular disease and diabetes, and such high-risk groups exhibit common metabolic features of low levels of Gln, NAD^+^, and overproduction of hyaluronic acid (HA).^159–161^ Levels of Gln and NAD^+^ cause dysregulation of SIRT1, a key negative regulator of the Hyaluronan synthase 2 (HAS2) gene.^162^ These metabolic alterations eventually lead to the overproduction of HA and Plasminogen activator inhibitor 1 (PAI-1) and the expansion of Tregs and myeloid-derived suppressor cells (MDSCs) populations. Therefore, Gln deficiency has led to immune dysfunction and HA overproduction in people at high risk of COVID-19. HA can activate STAT3 through PAI-1.^163^ Due to dysregulation of SIRT1, STAT3, and O-GlcNacylation induce hyaluronic acid storm through activation of HAS2. In addition, although SARS-CoV-2 vaccines have significantly reduced COVID-19 cases, cells are placed under intense oxidative stress conditions after SARS-CoV-2 infection, which promotes the consumption of Gln to synthesize glutathione.^164^ This process exacerbates Gln deficiency in high-risk populations and may induce metabolic dysfunction. At the same time, it can also cause STAT3 pathway inactivation and PAI-1 activation, leading to severe complications of COVID-19 in some people. Small clinical trials have shown that Gln supplementation reduces post-infection severity in patients with COVID-19.^165,166^ However, this part of the study needs to be expanded to more accurately assess the value of Gln in the treatment of COVID-19.

## Arginine (Arg)

### Arginine metabolism

Arginine, also known as L-arginine, is a raw material for protein synthesis and an intermediate product of the urea and nitric oxide cycles.^40,167^ Arginine is classified as a conditionally essential amino acid, and its requirement depends on developmental stage and health status. In humans, small intestinal epithelial cells convert Gln and glutamate to citrulline, which is then transported by the circulatory system to renal proximal tubular cells, where arginine is synthesized by arginine-succinate synthetase and arginine-succinate lyase in the urea cycle. Arginine synthesis is impaired when small intestine and kidney function is impaired, thus creating a dietary requirement for arginine. In other cell types, arginine synthesis by citrulline is very low but dramatically increases when inducible nitric oxide synthase (NOS) increases (Fig. 5). Under these conditions, citrulline, a byproduct of nitric oxide synthesis, can recover arginine via the arginine-citrulline pathway.^168^ Arginine is important for cell division, wound healing, and immune function.^169–171^ Arginine from proteins can be catalyzed by PAD enzymes to citrulline, a process called citrullination, which is part of the normal immune process. Another type of post-translational modification is methylation by arginine methyltransferases (PRMTs), in which arginine can be methylated to either monomethylated arginine or dimethylated arginine. Arginine methyltransferases can be divided into three following classes: Type I PRMTs (PRMT1, PRMT2, PRMT3, PRMT4, PRMT6, and PRMT8) catalyze the production of asymmetric dimethylarginine; Type II PRMTs (PRMT5 and PRMT9) catalyze the formation of symmetrical dimethylarginine; Type III PRMTs are currently the only known PRMT7, which produces only monomethylarginine.^172^ Arginine methylation usually occurs in the glycine and arginine-rich "GAR motif". Many arginine-methylated proteins have been shown to interact with DNA or RNA, and the arginine residue acts as an important hydrogen donor for the phosphate backbone.^173,174^ In addition, arginine methylation also affects protein–protein interactions involved in various cellular processes such as protein trafficking, signal transduction and transcriptional regulation.^173^

### Arginine in cancer

**Fig. 8:**
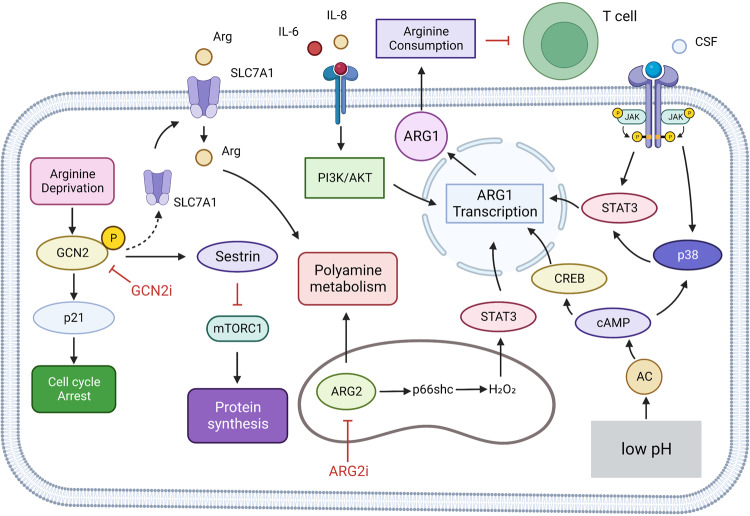
Arginine metabolism in tumor cells. Arginine depletion can increase the phosphorylation level of GCN2 in hepatocellular cancer cells, activate GCN, increase the expression level of SLC7A11 and increase the uptake of arginine. Activated GCN2 can also be mediated by p21 cell cycle arrest; GCN2 also increases protein synthesis by activating mTORC1 via sestrin. ARG2 in the mitochondria of melanoma cells increases transfer-promoting gene transcription via the p66SC-H2O2-Stat3 axis. Myeloid cells can promote intracellular p38 and ARG1 transcription by receiving tumor cell-derived CSF and activation of STAT3. In addition, low pH of tumor microenvironment also promoted ARG1 transcription through H^+^ activation of intracellular cAMP-CREB axis. IL-6 and IL-8 promote ARG1 transcription by activating the PI3K/AKT pathway. The arginine metabolism of myeloid cells with high expression of ARG1 was enhanced, and the arginine metabolism of T cells was inhibited, and the tumor immunity was inhibited. Created with BioRender.com. (The red blunt line represents inhibition)

Citrulline and aspartate can be converted to arginine in normal cells by arginine-succinate synthetase 1 (ASS1) and arginine-succinate lyase (ASL) in the urea cycle.^175^ Arginine-succinate synthetase 1 (ASS1) transcriptional repression occurs in various tumors, creating a dependence on external arginine and enabling arginine-deprivation therapy. Use of the arginine-depleting agent pegylated arginine deiminase ADI-PEG20 in GBM can increase nitric oxide (NO) synthesis and produce cytotoxic pernitrite, increasing the sensitivity of tumor cells to ionizing radiation and significantly enhancing the effect of radiotherapy on GBM.^176^ The combination of ADI with Palomid 529 or chloroquine showed a synergistic tumor inhibition effect in vitro. Combination with suberoylanilide hydroxamic acid (SAHA) can effectively control the growth of GBM xenografts.^177^ ASS1 is downregulated in hepatocellular carcinoma (HCC); therefore, arginine dystrophy is also present. Treatment of HCC cells with ADI-PEG20 downregulates the key enzymes of pyrimidine synthesis in the TCA cycle, carbamoyl phosphate synthetase 2, thymine synthase (TS), aspartate transcarbamylase and dihydrooratase (CAD) and malate dehydrogenase 1 (MDH-1) activities, making tumor cells more susceptible to 5-fluorouracil (5-FU). The effect of this synergistic treatment is ASS-dependent, and the activity of the enzymes mentioned above can be restored by transfection of ASS, eliminating the sensitivity of tumor cells to ADI-PEG20 combined with 5-FU treatment.^178^ Meanwhile, arginine deprivation promotes GCN2-dependent cycle arrest in HCC cells, and inhibition of GCN2 in arginine-deprived HCC cells promotes cellular senescence and increases the efficacy of senolytic compounds (Fig. 8).^179^ Ass-deficient prostate and pancreatic cancers have also been shown to be sensitive to ADI-PEG20, and ADI-PEG20 promotes cell death by inducing autophagy and apoptosis.^180,181^

The arginase isoenzymes arginase1 (ARG1) and arginase2 (ARG2) are abnormally upregulated in various cancers. ARG1 is mainly expressed in the cytoplasm of hepatocytes and plays a role in the urea cycle. In contrast, ARG2 is expressed in mitochondria of multiple tissues, with the most abundant expression in the kidney and prostate, and mediates arginine/ornithine balance.^182,183^ In neuroblastoma, overexpression of ARG1 can increase AKT and ERK phosphorylation and promote cell proliferation.^184^ In gastric cancer, serum factors IL-6 and IL-8 can stimulate CD45^+^CD33^low^CD11b^dim^ MDSCs to express ARG1 and ultimately inhibit CD8^+^ T cell function through the PI3K-AKT signaling pathway (Fig. 8).^185^ GM-CSF derived from breast cancer tumor cells can promote ARG1 expression in tumor-infiltrating myeloid cells through STAT3 and p38 MAPK signaling pathways, enhance the function of tumor-infiltrating myeloid cells, and promote the development of immunosuppressive TME (Fig. 8).^186^ Studies have also found that the expression level of ARG2 in malignant thyroid tumors is significantly higher than that in normal tissues, and ARG2 inhibition can reduce the expression level of AKT and promote tumor cell apoptosis.^187^

In head and neck squamous cell carcinoma (HNSCC), phosphorylated STAT3 can directly bind to the ARG1 promoter region of MDSCs to promote transcription, thereby contributing to the immunosuppressive effect of MDSCs.^188^ ARG2 has also been reported to promote melanoma tumor metastasis through the H_2_O_2_-STAT3 pathway (Fig. 8).^189^ Multiple tissue-derived tumor samples show a large number of Arg1-positive myeloid cells in the tumor microenvironment. In cancer patients, ARG1 is increased while L-Arginine is decreased in plasma samples. Administration of CB-1158 (a small molecule inhibitor of arginase) can slow tumor growth rates, block myeloid cell-mediated suppression of T-cell proliferation, and increase the number of tumor-infiltrating CD8^+^ T cells and NK cells in mouse models of multiple tumors.^190^ Piceatannol (PIC), another natural arginase inhibitor, can effectively inhibit TGF-β1 /TGF-β receptor type 1 (TGF-βR1) signaling pathway, limit M2-type macrophage polarization to regulate TME and inhibit CRC progression and metastasis.^191^ These results suggest that ARG is a potential target for tumor metabolism. Arginase inhibitors have been developed and experimentally evaluated in various tumors.

#### Protein arginine methyltransferases (PRMTs)

PRMTs are SAM-dependent enzymes that catalyze the mono- and di-methylation of peptidyl arginine residues. Many studies have shown that the activity of PRMTs is related to cancer stem cells (CSCs), which can self-renew and generate differentiated progeny. This is an important factor leading to tumor drug resistance, metastasis and recurrence. PRMT5 is highly expressed in breast cancer and chronic myeloid leukemia (CML) stem cells, and the knockdown of PRMT5 or the use of PRMT5 inhibitors can significantly impair the self-renewal capacity of CSCs.^192,193^ PRMT5 can also promote DVL3 expression, thereby driving Wnt/β-catenin signaling.^192^ In addition, PRMT1 is a key regulatory molecule that maintains the pluripotent state of progenitor cells.^194^ PRMT7 can promote the transcription of Oct4, c-Myc, Nanog, and Klf4 by regulating the histone methylation of miR-24-2 and miR-221 promoters and inhibiting the expression of miRNA.^195,196^ PRMT8 can also maintain ES cell diversity by inducing SOX2 expression.^197^ In breast cancer, arginine 21 (R21) and lysine 108 (K108) on mitochondrial ribosomal protein S23 (MRPS23) are methylated by methyltransferase 7 (PRMT7) and SET-domain-containing protein 6 (SETD6), respectively. Methylation of R21 promotes the polyubiquitination and degradation of MRPS23, which inhibits mitochondrial phosphorylation (OXPHOS) and increases ROS levels, thereby promoting breast cancer cell metastasis. On the other hand, K108 methylation cooperates with R21 methylation to maintain low levels of OXPHOS, which favors breast cancer cell survival.^198^ The use of the PRMTs inhibitor MS023 in TNBC induces an interferon response and exerts an antitumor effect.^199^ In GBM, PRMT2 participates in oncogene transcription by mediating H3R8me2a modification.^200^ PRMT5 expression levels are elevated and associated with poor patient prognosis. Also, PRMT5 knockdown impairs the self-renewal ability of GBM cells and promotes apoptosis.^201,202^ Administration of the PRMT6 inhibitor EPZO20411 can inhibit the arginine methylation of chromatin condensation regulator 1 (RCC1), block the tyrosine kinase 2 (CK2) -PRMT6-RCC1 signaling axis, inhibit the proliferation of glioblastoma stem cells (GSCs) and increase their sensitivity to radiotherapy.^203^ Targeting PRMT7 reduces glycine decarboxylase expression, leading to the reprogramming of glycine metabolism and production of methylglyoxal, impairing the self-renewal capacity of leukemia stem cells (LSCs) and slowing the progression of leukemia.^204^ Similarly, administration of the PRMT5 inhibitor PJ-68 in a CML mouse model with high expression of PRMT5 LSCs could deplete DVL3, thereby inhibiting Wnt/β-catenin signaling and significantly prolonging the survival time of a retroviral BCR-ABL-driven CML mouse model.^193^ These studies prove that PRMTs are potential targets for cancer therapy, and several PRMTs inhibitors are currently in clinical trials (Table 1).

### Arginine in disease

#### Wound healing

In the process of wound healing, arginine participates in the response of inflammatory factors through the arginine-NO pathway. In addition, ornithine and urea produced by arginase degradation of arginine are essential during this process and have a key role in the synthesis of collagen and polyamines.^171,205,206^ Arginine can promote fibroblast proliferation through GPRC6A-ERK1/2 and PI3K/Akt signaling pathways.^207^ Arginine can also increase monocyte migration and proinflammatory factor production in peripheral blood during the early stages of inflammation.^208^ In the later stage of inflammation, arginine can also inhibit the activity of immune cells and regulate immune status. In myeloid cells, activated nitric oxide synthase (NOS) and NO inhibit T lymphocyte function by interfering with IL-2.^209^ In summary, arginine and its metabolites are essential for wound healing and are involved in multiple stages of wound healing, including collagen formation, cell proliferation, and immune regulation.

Dietary arginine supplementation is the most convenient way and has multiple benefits for wound healing. Arginine supplementation enhances the body’s DNA synthesis.^210^ In colitis models, arginine supplements inhibit the expression of inflammatory factors and chemokines, suppress the inflammatory response, and promote the repair of injured tissues.^211^ Patients undergoing trauma/hemorrhagic shock have difficulty achieving wound healing due to reduced collagen synthesis. On the contrary, arginine supplementation significantly alleviates the above problems and increases wound strength.^212^ During diabetic wound healing, arginine supplementation can reverse the insufficient synthesis of NO and restore the concentration of NO in damaged tissues, promoting wound healing.^213^ Arginine has also been used to mitigate the risk of pressure ulcers, and supplementation of arginine in patients at high risk for pressure ulcers can significantly accelerate pressure ulcer healing.^214^

#### Alzheimer’s disease (AD)

Alzheimer’s disease (AD) is characterized by senile plaques and neurofibrillary tangles (NFTs) caused by amyloid-β and phosphorylated tau deposition. Advanced glycation end products (AGEs) modify proteins to cause their dysfunction. Glycosylation of the AMPK-γ subunit inhibits AMPK function, and arginine treatment protects AMPK-γ from glycosylation and increases AMPK phosphorylation in a mouse model of AD, thereby ameliorating AD disease.^215^ In patients with mild AD/cognitive impairment (MCI), a combination of L-arginine, HMG-CoA inhibitor simvastatin, and tetrahydrobiopterin to enhance the endothelial nitric oxide synthase (eNOS) pathway modestly increases cerebral blood flow and improves cognition.^216^ In addition, PRMT4-catalyzed asymmetric dimethylarginine (ADMA) has been reported to bind to NOS as a ligand, leading to NOS dysfunction, resulting in decreased cerebral blood flow and aggravating AD, which can be reversed by inhibition of PRMT4.^217^

#### Lung disease

Asthma is a variable, recurrent, long-term inflammatory disease of the respiratory tract. Imbalances in the metabolism of Arg and nitric oxide have been implicated in the pathophysiology of asthma. An analysis of plasma metabolic mass spectrometry in children with asthma showed that Arg, Lys, and Met levels were significantly decreased in the susceptible asthma group compared to the non-susceptible asthma group.^218^ Althoff et al. also showed significant increases in serum concentrations of asymmetric dimethylarginine (ADMA), enhanced inhibition of NOS, enhanced catabolism of Arg, increased levels of ornithine (Orn) and proline (Pro), and decreased Arg/Orn ratio in patients with asthma and obstructive sleep apnea (OSA).^219^ A controlled trial conducted by Liao et al. showed that adding L-Arginine to labeled asthma medications did not significantly reduce asthma exacerbations.^220^ This may be due to a marked increase in the activity of arginase induced by IL-4 and IL-13 and a marked increase in the downstream product putreamine in allergen-stimulated lungs.^221^ The addition of L-citrulline (a precursor of the L-arginine cycle and NO synthesis) to medications in obese asthmatic patients may assist in asthma control and improve fractional NO excretion (FeNO) levels.^222^

In chronic obstructive pulmonary disease (COPD), monocytes in lung tissue induce the transcription of PRMT7 through the NF-κB/RelA signaling pathway. High expression of PRMT7 promotes the methylation of RAP1A regulatory element histones and regulates monocyte adhesion and migration. Decreased expression of PRMT7 reduces monocyte recruitment to sites of lung injury.^223^

#### Cardiovascular diseases

Patients with hypercholesterolemia and vascular disease commonly have elevated asymmetric dimethylarginine (ADMA), which is associated with impaired NO synthesis and an early marker of endothelial dysfunction.^20^ ADMA is an endogenous nitric oxide synthase (NOS) inhibitor that can significantly reduce the synthesis of vasodilator NO, leading to the development of cardiovascular disease. The abnormal activity of PRMTs results in increased ADMA and MMA, which increases the risk of cardiovascular disease. Loss of PRMT7 in the heart reduces symmetrical dimethylation of β-catenin, enhances Wnt-β-catenin signaling, and promotes myocardial hypertrophy.^224^ PRMT1 is the main enzyme that catalyzes ADMA. It regulates gene activation by regulating histone methylation modification in the promoter region of myocartin. Ablation of PRMT1 can downregulate the expression of contractile genes such as myocartin and significantly reduce the contractility of the aorta and the traction force of vascular smooth muscle cells (VSMCs).^225^ Administration of the PRMT4 inhibitor TP-064 in a mouse model of atherosclerotic cardiovascular disease can induce a decrease in monocyte tumor necrosis factor-α (TNF-α) secretion, downregulate the gene expression of the glycogen metabolism-related protein G6pc in the liver, and reduce plasma triglyceride levels, exerting regulatory effects from inflammatory and metabolic pathways.^226^

Inhibitors targeting PRMTs are being developed and experimentally tested. Arg methylase inhibitors (AMIs), symmetric sulfonated urea, specifically inhibit PRMT activity and, in a rat model, cyclooxygenase-2 (COX-2) expression and suppress inflammation.^227^ MS023, another selective PRMT type I inhibitor, reduced ADMA, increased MAA and SDMA levels, and shifted PRMTs activity from type I to type II/III in cells treated with MS023.^228^

## Methionine (Met)

### Methionine metabolism

Met is an essential amino acid and a precursor of other amino acids such as cysteine (Cys) and taurine, as well as S-adenosyl-L-methionine (SAM) and glutathione (GSH). The backbone of Met biosynthesis is mainly derived from aspartate.^229^

Aspartate is first converted to homoserine through the reduction reaction of the β-aspartate semialdehyde terminal. The intermediate aspartate semialdehyde can be condensation with pyruvate to participate in the Lys biosynthesis pathway, and homoserine itself can also participate in threonine biosynthesis. The homoserine hydroxyl group is then activated by phosphate, succinyl, or acetyl groups, and the hydroxyl group is then replaced by cysteine, methyl thiol, or hydrogen sulfide by displacement reactions. It reacts with Cys under the catalysis of cystathionine-γ-synthetase to produce cystathionine, which is cleaved by cystathionine-β-lyase to form homocysteine. In reaction with free hydrogen sulfide, homocysteine is formed under the catalysis of O-acetyl-homoserine aminocarboxypropyl transferase. The reaction with methanethiol yields Met directly.^229^ Cysteine and homocysteine can be interconverted through the sulfur transfer pathway. This pathway includes both forward and reverse. The forward pathway is present in bacteria such as *Escherichia coli* and *Bacillus subtilis* and can transfer sulfhydryl groups from cysteine to homocysteine.^230,231^ The reverse pathway exists in organisms, including humans, to transfer sulfhydryl groups from homocysteine to cysteine.^232^ Even though Met is classified as an essential amino acid because of the reverse sulfur conversion pathway in humans, cysteine does not fall into this category.

In catabolism, Met is catalyzed by Met adenosine transferase (MAT) to SAM. As a methyl donor, SAM participates in various methyl transfer reactions and is converted to S-adenosylhomocysteine (SAH) in the reaction. Met can increase the intracellular concentration of glutathione, promote cellular REDOX regulation, and protect cells by binding to oxidative metabolites (Fig. 5).^233^

### Methionine in cancer

**Fig. 9:**
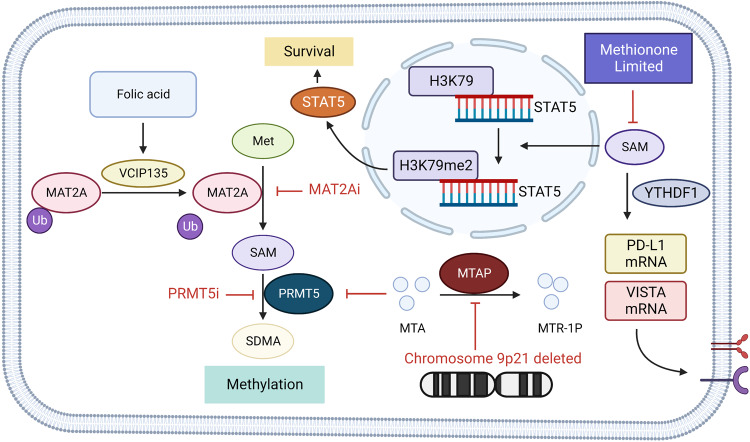
Methionine metabolism in tumor cells. In hepatocellular carcinoma, valosin-containing protein P97/P47 complex-interacting protein 1 (VCIP135) responded to folic acid signals to bind and stabilize MAT2A. In MTAP deficient cells, the MTAP substrate, MTA, accumulates and inhibits PRMT5 activity. Tumor cells can increase methionine intake through high expression of SLC43A2, competitive consumption of methionine in the environment, resulting in methionine deficiency in T cells. T cell methionine restriction can inhibit the normal methylation in cells, resulting in the transcription of STAT5 gene obstruction, affecting T cell survival and function. On the other hand, methionine metabolism inhibited PD-L1 and V-domain Ig suppressor of T cell activation (VISTA) immune checkpoint translation. Created with BioRender.com. (The red blunt line represents inhibition)

Met, as an essential amino acid, has an important role in tumor growth and metabolism. In addition to exogenous supply, the Met salvage pathway is the only Met source. This pathway requires the activity of methyladenosine phosphorylase (MTAP) as well as Met synthase (MS).^234^ MTAP is located in the periphery of the tumor suppressor cyclin-dependent kinase inhibitor 2 A (CDNK2A), and the co-deletion of the two genes occurs in ~15% of cancers and results in a highly aggressive tumor with a poor prognosis. These enzymes are often downregulated in malignant tumors, resulting in a strong dependence of the cells on Met intake from the external environment.^234,235^ In addition, Met is broken down by the Met cycle, in which Met adenosine transferase 2A (MAT2A) is upregulated and highly active in tumors.^236^ MAT2A has been proposed as a lethal target in MTAP null tumors. In the absence of MTAP, the substrate methyl thionyl adenosine (MTA) accumulates and acts as a selective inhibitor of PRMT5 to inhibit PRMT5 methylation activity,^237^ whereas MAT2A can produce the PRMT5 substrate SAM,^238^ allowing PRMT5 to exert its oncogenic effect. Valosin-Containing Protein P97/P47 Complex-Interacting Protein 1 (VCIP135) binds and stabilizes MAT2A in response to folate signaling in HCC and promotes tumor formation and progression in DEN/high-fat diet (HFD)—induced HCC mouse models (Fig. 9).^239^ Treatment with MAT2A inhibitors AG-24152 and AG-270 significantly reduces SAM levels, inhibits PRMT5 activity, and causes DNA damage and mitotic defects in tumor cells. Also, AG-270 showed a synergistic antiproliferative effect with the anti-mitotic drug taxane in vitro and in vivo.^240^ It has also been shown that MTA accumulation and secretion occur in MTAP-deficient GBM, but no significant MTA accumulation was detected in vivo because the stromal cells in the TME express WTAP and consume the MTA emitted by tumor cells and increase the activity of PRMT5. PRMT5 inhibitors GSK591 and ly-283 caused a broad inhibition of transcriptome splicing in GBM cells, particularly affecting the products of cell cycle genes and prolonged survival in the PDX mouse model.^241^

Because of its central role in methylation, Met is considered a candidate target for tumor therapy driven by ten-eleven translocation (TET), isocitrate dehydrogenase (IDH) proteins, methyltransferases, and other phenotypic modifiers. Anti-tumor effects of the Met-free diet were first reported in Walker-256 sarcoma-bearing Sprague-Dawley rats.^242^ Met restricted diet can reduce N6-methyladenosine (m6A) methylation and immune checkpoint translation, such as PD-L1 and V-domain Ig suppressor of T cell activation (VISTA) in tumor cells. In addition, it increases the number of tumor-infiltrating CD8^+^ T cells to inhibit tumor immune escape (Fig. 9).^243^ In AML, the level of H3K36me3 changes most dramatically before and after Met deprivation, and inhibition of STED2, the H3K36-specific methyltransferase, can reproduce most of the cytotoxic phenotypes produced by Met deprivation.^244^ In addition, tumor cells can consume a large amount of Met in the environment by highly expressing Met transporter SLC43A2, which can inhibit the Met metabolism of T cells in the microenvironment, resulting in the loss of dimethylation of histone H3K79me2 lysine 79 in T cells, low expression of STAT5, and inhibition of T cell function. Met supplementation or inhibition of SLC43A2 expression in tumor cells can reverse the above-mentioned functional suppression of T cells and activate tumor immunity (Fig. 9).^245^

The hepatocyte nuclear factor 4α (HNF4α) regulates sulfur amino acid (SAA) metabolism in the liver. Knocking down HNF4α in hepatocellular carcinoma impairs SAA metabolism, increases tumor cell tolerance to Met deprivation and sorafenib, and promotes tumor EMT. In contrast, restoring SAA metabolism alleviated the tumor phenotype resulting from HNF4α deficiency.^246^ These studies suggest that Met starvation not only suppresses tumor cell metabolism but also involves immune cells and that tumor cells themselves have complex preferential pathways to regulate Met metabolism. Therefore, more precise and focused treatment methods should be developed for tumor cell Met metabolism. Targeting STED2 and SLC43A2 provides new insights.

### Methionine in disease

#### Fatty liver disease

Nonalcoholic fatty liver disease (NAFLD) is a disease caused by abnormal metabolic pathways leading to the accumulation of triglycerides (TG) in the liver. Obesity and T2D mellitus are strong risk factors for NAFLD. HFD and Met and choline-deficient diet (MCD) can mimic human diseases’ histological and metabolic abnormalities and are commonly used to establish NAFLD mouse models. When evaluating the differences in the construction of the NALFD/NASH model between the two methods, it was found that the MCD diet could spontaneously lead to liver fibrosis within 2–4 weeks and significantly affect the expression of genes involved in liver fibrosis pathways. This effect of HFD was not observed until 24 weeks after insulin resistance, which resulted in less liver fibrosis.^247^ Clinical data show that Met levels are reduced in the early stages of NAFLD and that higher Met intake is inversely associated with fibrosis risk. Methyl donor supplementation reduces hepatic fat accumulation by activating the AMPK signaling pathway to increase fatty acid consumption.^248^ Also, SAM has a key role in regulating liver homeostasis, and reduced levels of SAM synthesis have been detected in various chronic liver injuries, such as NAFLD.^249^ SAM can alleviate fat accumulation and oxidative stress by promoting mitochondrial fatty acid β-oxidation and releasing TG.^249^

In alcoholic liver disease, long-term alcohol intake increases the levels of homocysteine and SAH, decreases the SAM/SAH ratio, and directly affects cellular methylation levels.^250,251^ At the same time, ethanol can directly affect the activities of MAT, BHMT, and other enzymes, interfere with Met metabolism, inhibit GSH formation, and weaken the antioxidant capacity of cells.^252^ SAM supplementation reverses GSH depletion, reduces mitochondrial DNA damage, and ameliorates steatosis and hepatocyte necrosis in ethanol-fed models.^253,254^ Moreover, betaine can similarly mitigate ethanol-induced liver injury by improving ethanol-induced fatty acid synthesis by targeting FA synthetases, peroxisome activator receptor γ (PPAR γ) coactivator 1, and hepatic sterol regulatory element binding protein (SREBP) − 1c.^255^ Methionine adenosyl-transferase α1 (MATα1) synthesizes SAM in the liver. As a transcription factor, MATα1 negatively regulates cytochrome P450 2E1 (CYP2E1) at the mRNA level. On the other hand, MATα1 directly interacts with CYP2E1 to promote the methylation of CYP2E1 at R379 site and degradation through the proteasome pathway. Patients with ALD have reduced levels of MATα1 and a reduced hepatocyte methylation/CYP2E1 ratio. MATα1 KO hepatocytes can also be detected with reduced methylation/CYP2E1 ratio, reduced mitochondrial membrane potential, increased ROS content, and are sensitive to ethanol and TNF-α-induced mitochondrial dysfunction.^256^ In addition, ethanol could activate kinase CK2 to phosphorylate MATα1 at Ser114, promote its interaction with PIN1 isomerase, inhibit MATα1 localization in mitochondria, and promote ethanol-induced mitochondrial dysfunction and fat accumulation. Blocking the interaction between PIN-1 and MATα1 reversed the alcohol-induced cytotoxic phenotype.^257^

#### Kidney disease

Autosomal dominant polycystic kidney disease (ADPKD) is a common monogenic disease characterized by the enlargement of renal cysts. In the ADPKD model, the levels of Met and SAM are increased, which induces the expression of Mettl3. Also, Mettl3 can increase c-Myc and Avpr2 mRNA modification, activate c-Myc and cAMP pathways, and accelerate cyst growth. A Met restricted diet may slow the progression of ADPKD.^258^ Kidney injury markers clusterin and cystatin c significantly decreased in the methionine restriction (MR) mouse injury model. Compared with the normal feeding model, the kidney inflammation genes such as Emr1, Nos2, and Tnfa were downregulated, and the degree of basophil aggregation was lower in the MR model. The renal fibrosis genes Fn1, Serpine1, Tgfb1, and Col1a1 were downregulated, and the degree of fibrosis was milder.^259^

#### Diabetes

Elevated levels of circulating Met, acetyl-aspartate, and Asn can be detected in T2DM and diabetic kidney disease (DKD). Also, elevated circulating Met levels can be used to predict the risk of developing diabetes.^260^ Met metabolism regulates Cys and endogenous hydrogen sulfide (H_2_S) levels. H_2_S inhibits glucose-induced insulin release in pancreatic β cells and insulin-stimulated glucose uptake in adipose tissue. Cystathionine γ-lyase (CSE) is a key enzyme in H2S synthesis, and the use of CSE inhibitors increases glucose uptake by adipocytes.^261^ MR can enhance insulin-stimulated phosphorylation of AKT and S6 and activate PI3K/AKT signaling pathway. Meanwhile, MR can also downregulate genes involved in an inflammatory response and immune cell infiltration, such as chemokine receptor (CCRs), chemokine ligand 7 (CCL7), IL-1β, IL-6, IFN-γ, and TNF-α.^262^ In summary, Met restriction (MR) can alleviate diabetes by interfering with glucose homeostasis, increasing insulin sensitivity and inflammatory response.

## Amino acid metabolism in the tumor microenvironment (TME)

Over recent years, more and more studies have shown that amino acids in different cells in the TME and their interactions affect tumor immunity and therapeutic effect. Amino acids, transporters, and metabolites participate in tumor immunity through metabolic reprogramming. In addition, specific amino acid deficiency or the immunosuppressive effect of certain amino acid metabolism can damage the function of immune cells, including effector T cells, in the tumor microenvironment. The function of T cells is closely related to the effect of immunotherapy, chemotherapy and other tumor treatments.^263,264^ This section will present the current advances in amino acid metabolism and immunity.

### BCAA

BCAAs have an important role in supporting immune cell function as carbon backbone providers in immune cells. A deficiency of BCAA impairs the immune function of lymphocytes and leukocytes.^265^ Leu depletion in T cells leads to restricted mTORC1 signaling and inhibits T-cell activation. A reduction in IFN-γ and IL-2 release from T cells was detected when T cells were co-stimulated with anti-CD3 and anti-CD28 using the Leu analog N-acetyl-Leu amide (NALA).^266,267^ Also, BCAA supplementation can increase CD8^+^ T cells to upregulate glucose transporter GLUT 1 in a FoxO1-dependent manner, increase glucose uptake and utilization, and enhance the antitumor activity of CD8^+^ T cells, having a synergistic role with anti-PD-1 therapy.^268^

There is a subset of immunomodulatory B cells in the TME with TGF-β1 as the main regulatory feature and expressing Leu-tRNA-synthase 2 (LARS2). This subpopulation of LARS B cells shows a preference for Leu metabolism, which can be induced by a Leu diet to promote mitochondrial NAD^+^ production and oxidative metabolism and recruit NAD^+^-dependent protein deacetylase SIRTUIN-1 (SIRT1) to participate in the regulation of LARS B cells. Depletion of LARS B cell subsets by LARS gene ablation or Leu depletion can inhibit immune escape in CRC.^269^

BCAA uptake is dependent on the type I amino acid transporter LAT, and mutations in SLC7A5 and SLC3A2, members of the LAT family, impair BCAA uptake by T cells and inhibit the proliferation and differentiation of Th1, Th17, and CTL cells.^41^ Slc3a2-dependent BCAA metabolism also has a key role in the physiological activities of Foxp3^+^ Treg cells, and either BCAA deprivation or SLC3A2 mutation can induce impaired activation of the mTORC1 pathway in Treg cells and inhibit the immunosuppressive function of Treg cells.^270^ Interestingly, a study on the correlation between amino acid metabolism and the immune microenvironment in LUAD (Lung adenocarcinoma) patients found that SLC7A5 expression was downregulated in various T cells, especially in effector T cells. However, high expression of SLC7A5 in tumor cells predicts reduced expression of immune-related genes, reduced immune cell infiltration, and poor efficacy of immunotherapy.^271^ Another bioinformatics analysis for breast cancer reached a similar conclusion.^272^ In addition, BCAT isoenzymes may serve as markers of tumor TME status. In non-malignant T cells, such as activated CD4^+^ T cells and CTL cells, BCAT c accounts for 50% of the total BCAT expression level, whereas this proportion is upregulated to 60% in T-cell lymphomas. Consistent with this phenomenon, the expression level of BCAT m is decreased in tumor tissues.^273^ In malignant gliomas, BCAT c gene expression has been reported to be positively correlated with M2-type macrophages and Treg markers,^274^ and GBM cells with high BCAT c expression can excrete BCKAs through monocarboxylate transporter 1 (MCT1). Increased uptake of BCKAs by M1 macrophages inhibits the phagocytic capacity of M1 macrophages and may therefore produce immunosuppression.^275^ However, positive correlations between BCAT c gene expression levels and infiltration levels of CTL, CD4^+^ T cells, macrophages, and dendritic cells have also been reported in colorectal and squamous cell carcinomas.^276^

### Aspartate

As a nonessential amino acid, cells can supplement Asp via the de novo synthetic pathway. The aspartate synthesis pathway requires the mitochondrial ETC to provide electron acceptors.^92^ Thus, aspartate is a limiting factor for growth under hypoxic conditions. When ETC is limited, cells rely on the amino acid transporter SLC1A3 for Asp uptake from the environment. Inadequate mitochondrial Asp production is an important cause of T cell dysfunction, and lack of aspartate inhibits nicotinamide purine dinucleotide (NADH) production, causing ER expansion and TNF release.^12^ In addition, Asp can promote the activation of hypoxia-inducible factor HIF-1α and inflammasome in M1 macrophages and increase the production of Asn and other metabolites. At the same time, Asn can also promote IL-1β secretion by M1 macrophages.^277^ Inhibition of aspartate aminotransferase in macrophages inhibits nitric oxide and IL-6 production by M1-type macrophages.^278^ However, in T cells, Asn can enhance the antitumor effect of CD8^+^ T cells by binding to the SRC family protein tyrosinase LCK and assisting LCK to phosphorylate at Tyr394 and 505, thereby enhancing the activity of Lck starved T cell receptor signaling pathway.^100^ In helper T cell 1 (Th 1), the electron transport chain complex I, the apple-aspartate shuttle, and citrate are required for aspartate synthesis and helper T cell proliferation.^279^

### Glutamine

Gln is the most abundant and versatile amino acid in the body. In general, the requirement for Gln by immune cells is similar to that of glucose.^280^ Gln can promote the proliferation of immune cells by activating ERK, JNK, and other proteins and increasing the transcription of cell proliferation genes.^125^ In addition, Gln can promote the expression of lymphocyte surface markers such as CD25 and CD71 and increase the production of cytokines such as IFN-γ and TNF-α.^281–283^ In the TME, Gln metabolic reprogramming is essential for the survival of tumor cells and immune cells, and there is competition for Gln uptake. For example, studies have found that triple-negative breast cancer (TNBC) competes for Gln uptake in the environment, limiting Gln metabolism in tumor-infiltrating T cells and inhibiting anti-tumor responses. In contrast, in models with GLS mutations, glutamate metabolism in tumor cells is restricted, which increases Gln concentration in the microenvironment and T-cell uptake and antitumor activity.^284^ A similar phenomenon has been observed in Gln-dependent clear cell renal carcinoma (RCC), which competitively depletes environmental Gln, causing local Gln depletion and IL-23 release from tumor-infiltrating macrophages, which further activates Treg function to suppress tumor immunity.^285^ Based on the above phenomena, researchers have developed therapeutic ideas to target Gln metabolism in tumor cells, increase the concentration of Gln in TME, and promote Gln metabolism in tumor-infiltrating cells.

Administration of the Gln synthetase inhibitor CB-839 promotes the differentiation and cytokine secretion of CD4^+^ Th 1 cells as well as CD8^+^ T cells while inhibiting the differentiation and co-function of Th 17 cells.^286^ This suggests that T cell subsets are heterogeneous for Gln metabolism.^287^ The currently developed JHU-083 is a prodrug of 6-Diazo-5-oxo-L-norLeu (L-DON) generation glutaminase inhibitor that selectively activates L-DON in the TME.^288^ Existing studies have demonstrated that L-DON and JHU-083 can extensively inhibit Gln metabolizing enzymes and activate AMPK and c-Myc to inhibit glycolysis, thereby stopping tumor metabolic activity, alleviating hypoxia, and increasing the concentration of Gln and glucose in TME.^289^ At the same time, the promotion of CD8^+^ T cell activation and recruitment and significant inhibition of MDSCs generation and recruitment were observed in models treated with L-DON and JHU-083.^290^

V-9302, an inhibitor of Gln transporter SLC1A5, increases intracellular ROS and autophagosome production. A preclinical study showed that V-9302 could selectively block Gln uptake by TNBC cells, promote the activation of CD4^+^ T cells and CD8^+^ T cells, and reduce the level of Treg in a TNBC mouse model. Interestingly, CD8^+^ T cells compensatory upregulate the Gln transporter ATB^+^/SLC6A14 to maintain their Gln uptake requirement after treatment with V-9302, but this phenomenon was not observed in tumor cells.^284^ In addition, PD-L1 upregulation was observed in tumor cells after V-9302 treatment, and the combination of V-9302 and anti-PD-1 antibody showed greater anti-tumor efficacy than either drug alone.^291,292^

### Arginine

Accumulating evidence has shown that arginine has an important role in regulating the function of immune cells. Human Burkitt B lymphocytes require an adequate arginine concentration for proliferation and maturation.^293^ Supplementation of the diet of high-risk surgical patients with an immune-enhancing diet (IED) containing arginine reduces the incidence of infection and increases macrophage phagocytosis, IL-2 expression, and CD4^+^ T cell numbers.^294,295^ However, some clinical trials have shown no benefit,^296,297^ so the value of arginine supplementation needs to be further tested. Although the effect of arginine supplementation remains to be tested, many studies have demonstrated that the downregulation of TCR receptor complex subunit CD3ζ in T cells cultured under arginine-restricted conditions leads to the restriction of T cell proliferation.^298–300^ However, the addition of citrulline, a precursor for arginine synthesis, can promote the expression of this molecule by prolonging the half-life of CD3ζ.^301^ In addition, arginase 1 (Arg1) administration to activated T cells leading to arginine starvation can block T cell glycolysis.^302^ Myeloid cells can activate their own Arg 1 expression in response to tumor-derived GM-CSF stimulation through activation of STAT3, p38, and cAMP signaling pathways, and Arg 1-expressing myeloid cells depleting environmental arginine inhibits T cell function.^186^ However, T cells have a mechanism to combat arginine deficiency: when confronted with arginine deficiency caused by Arg1-expressing myeloid cells, T cells increase arginine biosynthesis by up-regulating ASS1 expression.^303^

Arginase 2 (Arg 2) is a regulator of activated T cells, and Arg 2-deficient or Arg2^−/−^ CD8^+^ T cells exhibit enhanced cytotoxicity and synergistic effect with anti-PD-1 antibody in inhibiting tumor growth.^304^ In melanoma, Tregs expressing Arg 2 were detected, and Arg 2 inhibited mTOR activity and enhanced the immunosuppressive activity of Tregs.^305^ At the therapeutic level, therapeutic vaccines targeting Arg 1 have been shown to activate antitumor immunity in a variety of syngeneic mouse tumor models such as lung cancer, melanoma, and colon cancer, and the combination of Arg 1 vaccine with anti-PD-1 antibody can increase T cell infiltration, inhibit myeloid cell function, and increase the ratio of tumor-infiltrating M1/M2 macrophages.^306^ A similar conclusion was reached in the study of arginase inhibitor CB-1158. CB-1158 could block myeloid cell-mediated inhibition of T cell proliferation, increase the number of tumor-infiltrating CD8^+^ T cells and NK cells, and increase the expression of inflammatory factors and interferon-α, thereby changing the immune microenvironment to promote inflammation and reduce tumor immune escape, inhibiting tumor cell proliferation.^190^

On the other hand, arginine methylases (PRMTS) are widely expressed enzymes that catalyze the arginine methylation of proteins. Among them, type I PRMTs (PRMT1, PRMT2, PRMT3, PRMT4, PRMT6, and PRMT8) catalyze asymmetric dimethylated arginine to regulate DNA damage and transcriptional regulation, which is closely related to the occurrence and development of tumors. Applying type I PRMT inhibitor GSK3368715 can inhibit PRMTS-mediated epigenetic modification of IFN genes, increase the response of IFN genes to immune signals, and reduce the expression of VEGF in immunosuppressive cells. In anti PD-1 resistant T cell rejection models, the application of type I PRMT inhibitors PT1001B or GSK3368715 can increase the number of tumor-infiltrating T cells and increase the efficacy of anti-PD-1 therapy.^307,308^

### Methionine

Met metabolism is involved in a variety of cellular functions, including REDOX, methylation, and immune regulation. A second group of innate lymphoid cells (ILC2s) has a key role in type II immune response. Met metabolism is critical for regulating the function of ILC2s. Blockade of Met metabolism or loss of STAT3 significantly inhibits ILC2s function.^309^ Recently, Met restricted diet (MRD) has been reported to have an important role in anti-tumor immune regulation. MRD inhibited SAM-induced m6A methylation and translation of immune checkpoints such as PD-L1 and V-domain Ig suppressor of T cell activation (VISTA) in various mouse tumor models such as colorectal cancer and sarcoma. It also increased the number and toxicity of tumor-infiltrating T cells and enhanced antitumor immune responses.^243^ Moreover, inhibition of m6A-specific binding protein YTHDF1 can enhance tumor immunity like MRD.^243^ In EBV (Epstein-Barr virus)-infected tumors such as Burkitt’s lymphoma, Met metabolism helps shape B-cell immortalization required to regulate EBV-latent genes. The passage of MRD impairs Epstein-Barr virus-driven B-cell immortalization and exposes EBV antigens on the surface of Burkitt’s lymphoma.^310^

Tregs are characterized by high Met uptake and SAM use. Met metabolism is also essential for Treg survival after IL-2 deprivation, and solute carrier protein SLC43A2 plays a key role in Met uptake and maintenance of Treg growth activity.^311^ High expression of SLC43A2 and high activity of Met adenosine transferase 2A (MAT2A) in tumor cells imply vigorous Met metabolism and competitive inhibition of Met metabolism, STAT5 expression levels, and antitumor immune function in CD8^+^ T cells.^245,312^ Meanwhile, TAMs with high MAT2A expression also showed strong Met metabolic activity, increased histone H3K4 methylation level and receptor-interacting serine/threonine protein kinase 1 (RIPK1) gene expression.^313^ Several therapeutic approaches targeting Met metabolism, including Met restriction, MAT2A inhibitors IDE397 and AG-270, are in clinical trials, and their therapeutic effects need to be verified.

In summary, how to specifically inhibit the amino acid metabolism of tumor cells and immunosuppressive cells in the microenvironment, and enhance the amino acid metabolism of anti-tumor immune cells such as CD8^+^ T cells, is an urgent problem to be solved. One idea is to enhance the activity of amino acid metabolism of CAR-T cells by adding cytokines that promote the expression of transporters related to amino acid metabolism, such as SLC1A5, SLC3A2 and SLC7A5, or directly importing transcripts encoding these AATs into T cells. Furthermore, developing small molecule inhibitors targeting BCAA transporters in tumor cells and immunosuppressive cells allows CD8^+^ T cells to obtain amino acid metabolic advantages in the immune microenvironment and enhance the anti-tumor effect.

## Targeted therapies and clinical research of AA metabolism (tentative)

In the above modules, we introduced the mechanisms of amino acids, related metabolic enzymes, and metabolites related to the occurrence and development of diseases. In addition, investigators are exploring therapeutic strategies to address this metabolic feature of the disease. Therefore, this section focused on the progress of clinical trials for treating amino acid metabolism in diseases.

AXA1125 and AXA1957 are oral endogenous modulator (EMM) compositions. AXA1125 contains five amino acids (Leu, iLe, valine, arginine, and Gln) in specific ratios and the amino acid precursor N-acetylcysteine (NAC), while AXA1957 is composed of five amino acids, Leu, iLe, arginine, Gln, and serine, as well as carnitine and NAC. A multicenter, single-blind, placebo-controlled, randomized clinical study (NCT04073368) assessed the effect of AXA1125 and AXA1957 on nonalcoholic fatty liver disease (NAFLD). Patients were treated with 16 weeks and magnetic resonance imaging (MRI)-proton density fat fraction [MRI-PDFF] and homeostasis model assessment of insulin resistance [HOMA-IR]) and homeostasis model assessment of insulin resistance (HOMA-IR) fibro-inflammation markers (alanine aminotransferase [ALT], corrected T1 [cT1], keratin-18 [K-18] M65, and N-terminal type III collagen pro-peptide [Pro-C3]) was applied. The results showed that the biological activity of AXA1125 was greater in patients with T2D, compared with placebo. The MRI-PDFF (−31.2% vs.−8.3%), ALT (−34.6% vs. −13.9%) and cT1 (−105.1% vs. −42.7 ms) of AXA1125 decreased more significantly. By week 16, a larger proportion of AXA1125-treated subjects (35–40%) than those in the placebo group (8–25%) reached clinically relevant thresholds for MRI-PDFF reductions of ≥30%, ALT reductions of ≥17 IU/L, and cT1 reductions of ≥80 ms.

Moreover, a phenotypic study on human primary macrophages and stellate cells suggested that AXA1125 can inhibit lipopolysaccharide (LPS)-induced TNF-α expression in M1 macrophages and increase the secretion of anti-inflammatory chemokine C-C motif ligands by M2 macrophages, as well as reduced Pro-C3 and HSP47 expression in HSC.^314,315^ Both compositions have demonstrated multi-target activity in NAFLD and are worthy of further continuing clinical trials.^316^

Another Phase 2 study evaluating AXA1125 for the treatment of fatigue after COVID-19 infection has been completed, and a clinical study evaluating the safety, efficacy, and tolerability of AXA1125 in the treatment of nonalcoholic steatohepatitis (NASH) is ongoing.

### BCAT

The use of BCAT1 Inhibitor 2 in the NAFLD model can inhibit the activation of JNK and AKT signaling pathways and BCL2/Bax/Caspase axis induced by oleic acid, alleviate lipid accumulation, and inhibit mitochondrial ROS formation and apoptosis.^317^ However, BCAT Inhibitor is currently in preclinical studies, and their clinical value has yet to be verified (Table 1).

### Branched chain keto acid dehydrogenase kinase (BCKDK)

Sodium phenylbutyrate targets BCKDK and is an accelerator of BCAA catabolism. In clinical trials on insulin resistance and type 2 diabetes, phenylbutyrate could significantly improve peripheral insulin sensitivity (ΔRd:13.2 ± 1.8 vs. 9.6 ± 1.8 µmol/kg/min, *p* = 0.02). Plasma BCAA levels and glucose levels were also decreased (Table 1).^318^

### LAT1

The amino acid transporter LAT1 (SLC3A2/SLC7A5) is a kind of cancer cell-specific transporter expressed in various sources of cancer, and the high expression level of LAT1 is closely related to the poor prognosis of patients. α-methyl aromatic amino acids are LAT1 specific, and 18F-labeled 3-fluoro-l-α-methyl-tyrosine (FAMT) has been used as LAT1 specific probe for cancer detection. JPH-203, a LAT1-specific inhibitor designed based on LAT1 ligands, has a strong affinity and does not show obvious toxicity in preclinical studies. In phase I clinical trials, JPH-203 showed excellent inhibition of solid tumors and was well tolerated.^319^ Phase II clinical trials are currently underway. In addition to JPH-203, other drugs currently in preclinical and clinical trials targeting LAT1 include IPA-131, QBS-10072S, TLX101-CDx, 124I-ACD-101, 211At-TLX-102, OKY-034, [18F] NKO-028 (Table 1).^320,321^

### ASNase

Asn is a mature target for amino acid depletion therapy in tumors. Most compounds that target tumor metabolism (methotrexate, 5-fluorouracil) fail to distinguish between tumor tissue and rapidly differentiating epithelial tissue (skin, bone marrow),^4,322^ whereas therapies targeting the specific amino acid dependence of tumor cells are cell-selective, such as leukemic blasts that are selectively dependent on Asn, the use of bacterial-derived ASNase in pediatric ALL has significantly improved the cure rate.^102^ Pegaspargase and Calaspargase pegol are two ASNase-targeted drugs that have shown good results in treating hematological tumors. In a controlled study, patients (1–21 years of age) with newly diagnosed ALL or lymphoblastic lymphoma were given pegaspargase (2500 IU/M2, once induction, 15 doses every 2 weeks starting at week 7) or calaspargase pegol (2500 IU/M2, once induction, 10 doses every 3 weeks starting at week 7). Both drugs showed significant antitumor activity, with serum asparaginase activity (SAA) ≥ 0.1 UI/ml (considered therapeutic) in ≥95% of patients in both groups after 18 days of treatment and 25 days after treatment. More patients receiving calaspargase pegol had SAA ≥ 0.1 UI/ml (88% vs. 17%; *p* < 0.001). Moreover, in 230 patients, 99% of those receiving pegaspargase and 95% of those receiving calaspargase and pegol achieved complete remission. In terms of prognosis, the 5-year event-free survival (EFS) was 84.9% (SE ± 3.4%) for pegaspargase and 88.1% (SE ± 3.0%) for calaspargase pegol. Treatment with both drugs achieved similar nadir SAA and survival outcomes. Therefore, it is considered that the dosing strategy can be further optimized (Table 1).^323^

In addition to the above two drugs, asparaginase-targeting drugs include OP-01, JZP-458, ERY-001, and PF-690. JZP-458 was well tolerated in phase I clinical trials.^324^ More recently, the drug has been approved for phase II/III clinical experiments showing good effectiveness and safety (NCT04145531) (Tables 1, 2).^325^

### Argininase

The presence of arginine-succinate synthetase 1 (ASS1) deficienct tumors is arginine-dependent, thus enabling arginine-deprivation therapy. Pegylated arginase has potential arginine degradation and antitumor activity. After intravenous administration of pegylated arginase, arginine can be metabolized to ornithine and urea, reducing plasma arginine levels. Pegylated arginine arginase (PEG-BCT-100) showed promising tumor suppressive activity, survival advantage, and safety against advanced HCC in phase I clinical trials of combination chemotherapy (oxaliplatin and capecitabine).^326^ A phase II clinical trial is currently underway (NCT03455140). However, there is a bottleneck in this therapy. Externally introduced arginase analogs can inhibit T cells’ antitumor activity by reducing the arginine level in the tumor microenvironment, just as the tumor or immunosuppressive cells expressing arginase 1 can. An alternative therapeutic approach that has been developed on the basis of this problem is the use of arginase-1 peptide vaccines that activate T cells to target and recognize cells expressing arginase 1. Recent clinical trials showed good safety of arginase 1 peptide vaccine in patients with refractory solid tumors (NCT03689192).^327^ yet, its effectiveness remains to be further tested (Table 1).

### Arginine deiminase (ADI)

ADI is an enzyme that catalyzes the interconversion of arginine and citrulline. As an arginine degradation tool, its analogs were investigated in treating tumors with arginine-succinate synthetase (ASS1) mutations and/or arginine-succinate lyase (ASL). Pegylated arginine deiminase (ADI-PEG-20), which duplicates arginine and increases tumor stress and cytotoxicity, increased the number of tumor-infiltrating T cells in phase I studies and was safe in combination with anti-PD-1 antibody, but with an increased risk of neutropenia.^328^ ADI-PEG-20, in combination with pemetrexed and cisplatin (ADIPEMCIS), was well tolerated in another phase I study of recurrent high-grade glioma (HGG) (NCT02029690).^329^ The role of ADI-PEG-20 in HGG warrants further investigation. In hepatocellular carcinoma (HCC) studies, ADI-PEG-20 has been shown in early clinical trials to make HCC animal models and patients more sensitive to FOLFOX chemotherapy through arginine depletion. However, a recent large global, multicenter phase II study of HCC showed that ADI-PRG-20 combined with 5-fluorouracil, leucovorin, and oxaliplatin (mFOLFOX6) was associated with an ORR of 9.3% versus 8.15% with FOLFOX4. There was a significant difference in PFS (3.8 months vs. 2.93 months), although the improvement in OS (14.4 months vs. 6.4 months) was exciting. Still, authors suggested that it is more likely to be due to the short median follow-up time and the high proportion of Censored patients. Limited treatment efficacy and low response rates with this combination led to the early termination of the study. Despite the early termination of the trial, it is interesting to note that 13 of 140 patients had a median duration of response of 10.2 months, indicating that these patients benefited from combination therapy and that exploring the mechanisms of this benefit should be the focus of future research.^239^ Another phase II/III study evaluating ADI-PEG-20 for cisplatin and pemetrexed in patients with malignant pleural mesothelioma is ongoing and has completed recruitment (NCT02709512) (Table 1).

### Arginine methyltransferases (PRMTs)

Type I PRMTs catalyze asymmetric dimethylation of arginine, which is associated with cancer. The overexpression of arginine methyltransferase 5 (PRMT5) in solid and hematological tumors leads to the elevation of the methylation level of arginine residues on functionally related proteins in tumor cells, which affects cell cycle regulation, mRNA splicing, cell differentiation, signal transduction, and other physiological processes. Current studies are exploring PRMT5 inhibitors as a treatment for PRMT5-dependent tumors. The PRMT5 inhibitor PF-06939999 inhibited the proliferation of non-small cell lung cancer (NSCLC) in cells and animal models and dose-dependent reduced symmetrical dimethylarginine (SDMA) levels.^330^ Another PRMT5 inhibitor, JNJ-64619178, has shown long-term PRMT5 inhibition and potent anti-tumor effect in lung, pancreatic, and hematological tumors. A phase I clinical trial is ongoing to evaluate JNJ-64619178 in advanced solid tumors (NCT03573310). GSK3368715 is a reversible type I PRMTs inhibitor that synergistically inhibits tumor growth when combined with PRMT5 inhibitors. Metabolite 2-methylthiophosphate is an endogenous inhibitor of PRMT5. Deletion of the key catalytic enzyme methylthioadenosine phosphorylase (MTAP) gene is associated with the sensitivity of GSK3368715,^331^ and a current phase II clinical study of GSK3368715 in breast cancer has been enrolled (NCT04676516) (Table 1).

### Glutaminase (GLS)

GLS supports tumor Gln synthesis. In preclinical studies, telaglenastat (CB-839), a Gln synthetase inhibitor, promoted the function of CD4^+^ Th 1 cells and CD8^+^ T cells, and inhibited the function of Th 17 cells.^286^ It also induced the regression of PI3KCA-mutant tumors in xenograft models in combination with 5-fluorouracil (5-FU).^145^ Telaglenastat, a glutaminase inhibitor, was also evaluated in combination with everolimus (TelaE) in a phase I trial of advanced/metastatic renal cell carcinoma (mRCC), and TelaE therapy was well tolerated in patients previously treated with TKIs and checkpoint inhibitors (NCT03163667). The median PFS was improved compared with placebo (PboE) (3.8 months vs. 1.9 months).^146^ Currently, a phase II trial evaluating telaglenastat in cervical, prostate, and metastatic cancer is underway (NCT04824937; NCT05521997).

Another glutaminase inhibitor, JHU-083, extensively inhibits Gln-metabolizing enzymes and increases the concentrations of Gln and glucose in the TME by inhibiting glycolysis, relieving the hypoxic state.^289^ In addition, CD8^+^ T cell activation and recruitment were increased in the JHU-083 treatment model, and MDSCs generation and recruitment were significantly inhibited.^290^ A phase I/II trial is currently underway to evaluate the efficacy of JHU-083 in advanced solid tumors (NCT04471415) (Table 1).

### SLC1A5

The amino acid transporter SLC1A5 (ASCT 2) is highly expressed in various tumor tissues and is associated with poor prognosis of cancer. MEDI7247 is a novel antibody-drug coupling compound (ADC) that couples an ASCT 2 human monoclonal antibody site to a dimer of peroxbenzodiazepine (PBD). In preclinical studies, the drug has shown strong anti-tumor activity and survival advantage in AML, DLBCL, cALL, and Burkitt lymphoma tumor models.^332^ Currently, phase I trials have been completed to evaluate the efficacy of MEDI7247 in treating ASCT2-positive hematological malignancies and advanced solid tumors (NCT03106428; NCT03811652). Another Gln transporter SLC1A5 inhibitor, V-9302, selectively blocked Gln uptake by TNBC cells, promoted the activation of CD4^+^ and CD8^+^ T cells, and reduced Treg levels in a TNBC mouse model (Table 1).^284^

### MAT2A

Methylthioadenosine phosphorylase (MTAP)-deficient tumors account for ~15% of solid tumors, including ~15% of NSCLC, 28% of esophageal cancer, 26% of bladder cancer, and 10% of esophagogastric cancer. In MTAP null tumors, inhibition of Met adenosine transferase 2A (MAT2A) inhibits Met synthesis of SAM, thereby inhibiting tumor growth. MAT2A has been proposed as a therapeutic target in tumors with MTAP gene deletion.^333^ AG-270 is an oral selective MAT2A inhibitor that selectively inhibits the proliferation of MTAP null cells and effectively reduces the level of SAM in tumor cells in tissue and xenograft tumor models.^334^ A phase I study is ongoing to evaluate AG-270 in advanced solid tumors and lymphomas (NCT03435250). IDE-397 is another MAT2A inhibitor with low hepatotoxicity and high solubility that has shown potent modulation of SAM and symmetric dimethyarginine (SDMA) in preclinical studies. A phase I study to evaluate IDE-397 in solid tumors is currently underway (NCT04794699) (Table 1).

### METAP

Met aminopeptidase (METAP) is a kind of cytoplasmic enzyme, metal catalytic protein hydrolysis N end Met residue in the newborn. This enzyme has a key role in angiogenesis and is essential for progressing diseases such as solid tumors and rheumatoid arthritis.^335,336^ First reported as the first reversible METAP inhibitor (METAPi) in 2003, LAF389 is a natural benzimide compound with dual METAP 1i and METAP 2i activities.^337^ Although all cell types responded to this inhibitor in vitro and showed promising therapeutic effects in vivo,^337^ phase I clinical trials for treating advanced solid tumors ultimately failed due to cardiovascular toxicity and wide variability in patient responses.^338^ A-357300, which was subsequently developed, has shown anticancer activity in mouse models of cancer, sarcoma, and nervous system tumors,^339,340^ and another candidate, METAPi A-800141, which was selected based on affinity selection based on mass spectrometry, has better selectivity and efficacy than A-357300.^341,342^ A-800141 has demonstrated anti-angiogenic and anti-tumor activity in multiple xenograft models, including neuroblastoma, colon cancer, prostate cancer, and B-cell lymphoma.^343^ Evexomostat (SDX-7320), which irreversibly binds METAP2 and regulates insulin, leptin, and adiponectin downstream, was one of the first drugs developed for cancer patients with metabolic complications. The efficacy of evexomostat in inhibiting the angiogenic proteins FGF (Fibroblast growth factors) and VEGF (Vascular endothelial growth factor) was validated in a phase I clinical trial in advanced solid tumors (NCT02743637). Enrollment is ongoing for phase II trials evaluating Evexomostat in metastatic breast cancer and in patients with diabetes (NCT05455619; NCT05570253) (Table 1).

## Conclusion and future perspective

Amino acids metabolism affects multiple levels of cell metabolism and many cell processes, from protein synthesis to epigenetic regulation. These physiological processes are closely related to maintaining cell homeostasis and normal function. Thus, abnormal amino acid metabolism can contribute to disease development.^344^ Herein, we discussed physiological, and metabolic patterns of BCAAs, Asp, Gln, Arg, Met metabolism, and the role of various amino acids and their related enzymes and products in disease.

BCAA includes Leu, iLe, and valine, and all three amino acids participate in the citric acid cycle by producing acyl-CoA derivatives via branched-chain amino acid transferase (BCAT), branched-chain α-keto acid dehydrogenase (BCKDH), which in a subsequent series of reactions produce acetyl-CoA.^45^ BCAA has an important role in tumor and metabolic diseases as an essential amino acid. For example, in PDAC and NSCLC, which share the same genetic mutation background (KRAS and p53 mutations), BCAA requirements are high but significantly different. Yet, the exact difference in BCAA requirement between tumors is still not fully understood. Existing evidence suggests that CBP and SIRT4 can promote the ubiquitin-proteasome degradation of BCAT2 by acetylation of BCAT2 at the K44 site in PDAC.^53^ This reveals a novel BCAA metabolism inhibition mode in the context of KRAS inhibition of BCAT2 ubiquitination degradation, explaining the preference for PDAC amino acid metabolism. However, on the other hand, studies on the interaction of different cells in the microenvironment have found that CAFs cells have a high metabolism of BCAA and provide BCKA to PDAC cells to assist tumor cells in BCAA metabolism.^57^ In addition, the phagocytic activity of macrophages exposed to BCKA is inhibited.^275^ This provides an idea for treating PDAC by targeting the BCAA metabolism of CAF cells in the tumor environment and also proves that the tumor and various cells in the environment communicate with each other as a whole. Subsets with different preferences for BCAA metabolism have also been found in breast cancer, and further studies are needed to determine whether this is due to differences in tumor cells or the involvement of other cells in the microenvironment.^55,59^ BCAA metabolism is equally important for the function of proinflammatory CD4^+^ and CD8^+^ T cells and immunosuppressive regulatory Treg cells,^41,265–272^ which plays key role in metabolic diseases, liver and kidney diseases.^10,11,65–84,345,346^ Currently, drugs targeting BCAA uptake-dependent amino acid transporter LAT1 and metabolic enzymes BCAT and BCKDK are in trials.^317–321,347^

Aspartate is a nonessential amino acid, but it is also an intrinsic limiting factor in the growth of some tumors.^92,93^ The transamination product of Asp, asparagine (Asn), is more permeable than Asp, but when cellular asparaginase activity is inhibited, Asn cannot be converted to Asp.^93^ Bladder cancer cells lack asparaginase, leading to dysfunction in converting Asn to Asp. Under hypoxia, the mitochondrial ETC is inhibited, and energy synthesis and Asp synthesis are limited, which leads to the dependence of tumor cells on the environmental uptake of Asp. The amino acid transporter SLC1A3 has an important role in maintaining Asp concentrations inside tumor cells and antagonizing the therapeutic effects of ASNase.^94^ SLC1A3 inhibitors can promote cell cycle arrest and apoptosis and counteract SLC1A3-induced ASNase resistance.^94^ Moreover, SLC1A3 is mainly expressed in brain tissue, and its high expressions in some solid tumors, such as clear cell renal cell carcinoma, papillary renal cell carcinoma, hepatocellular carcinoma and gastric adenocarcinoma, may make SLC1A3 inhibitors a solution to ASNase resistance. Another glutaminase inhibitor, JHU-083, extensively inhibits Gln-metabolizing enzymes and increases the concentrations of Gln and glucose in the TME by inhibiting glycolysis, relieving the hypoxic state.^289^ One limitation of ASNase therapy, which promotes tumor cell death by systematically lowering the asparagine concentration, is that it impairs ASN requirements in normal immune cells.^99,100^ This may explain the immune-related side effects of ASNase drugs and limit the application of ASNase in cancer treatment. Therefore, limiting Asn metabolism in tumor cells and immunosuppressive cells in the TME and protecting and promoting Asn metabolism in anti-tumor immune cells should be a problem to be solved. In treating solid tumors, developing more tumor-targeting ASNase is one aspect, and the SLC1A3 inhibitor mentioned earlier also provides an idea to solve this problem.

Gln is extensively consumed by intestinal, renal, immune, and tumor cells.^122–124^ The key Gln-regulated enzyme GLS and the amino acid transporter SLC1A5 are regulated by the oncogene c-Myc.^128^ Meanwhile, SLC1A5 expression was also regulated by STAT3 in AML cells. Gln depletion therapy can inhibit Gln metabolism in tumor cells, but in colon cancer, it has been observed that Gln depletion promotes the expression of the aspartate/glutamate transporter SLC1A3 in tumor cells, increasing the intracellular glutamate concentration and promotes Gln synthesis.^131^ In addition, the cystine/glutamate antiporter SLC7A11/xCT also has an important role in cellular Gln metabolism. Because SLC7A11 exchanges intracellular glutamate with extracellular cystine, the intracellular glutamate concentration decreases, which leads to more Gln uptake and increased glutaminase activity and making these cells dependent on external Gln. According to the TCGA database, The expression levels of SLC7A11 mRNA in Cervical cancer (CESC), Cholangiocarcinoma (CHOL), Colonic adenocarcinoma (COAD), Esophagus cancer (ESCA), Head and neck squamous cell carcinoma (HNSC), chromophobe kidney cell carcinoma (KICH), Clear cell carcinoma of kidney (KIRC), Papillary cell carcinoma of the kidney (KIRP), Liver cell carcinoma (LIHC), Lung adenocarcinoma (LUAD), Squamous cell carcinoma of the lung (LUSC), Adenocarcinoma of the pancreas (PAAD), Rectum adenocarcinoma (READ), Sarcoma (SARC), Cutaneous melanoma (SKCM), Stomach adenocarcinoma (STAD), and Endometrial carcinoma of the flesh (UCEC) were significantly higher than those in adjacent normal tissues. These features suggest that SLC7A11 may serve as a promising target for cancer metabolism. Glutaminase (GLS), as a key enzyme in tubular aminamide metabolism, has also received extensive attention. CB-839, an inhibitor targeting GLS, has shown good tumor inhibition activity, tolerance, and safety in preclinical studies and phase I clinical trials in solid tumors.^145,146,286^

Cells lacking arginine-succinate synthetase 1 (ASS1) are arginine-dependent. ASS1 expression is downregulated in CHOL, GBM, KICH, KIRC, KIRP, and LIHC tumor categories, suggesting the feasibility of arginine depletion therapy. Analogs targeting arginase and arginine deiminase, the enzymes involved in arginine depletion, have been developed. Using the arginine deiminase analog ADI-PEG-20 in hepatocellular carcinoma and glioblastoma has demonstrated antitumor activity in vitro and in xenograft models and demonstrated safety and efficacy in a phase I clinical trial.^328^ Although setbacks occurred in the phase II study, the combination of ADI-PEG-20 and mFOLFOX6 chemotherapy regimen did not show a significant therapeutic advantage for hepatocellular carcinoma,^239^ but there are a small number of subjects who benefit from this regimen. Studying the mechanism of benefit in this group of subjects to seek the precision of treatment should be the next problem to be solved. Pegylated arginase PEG-BCT-100 combined with oxaliplatin and capecitabine has shown satisfactory therapeutic efficacy and safety in phase I clinical trials in solid tumors.^326^ In addition, the role of arginine methyltransferase (PRMT) in the regulation of tumorigenesis and development has also received extensive attention, and a variety of PRMT inhibitors have shown good anti-tumor activity in vitro and animal models, and a variety of drugs targeting type I PRMT and PRMT5 have been tested.^330,331^

The status of Met as an essential amino acid and its role in the transmethylation process predestines the cells’ dependence on Met metabolism. Methythioadenosine phosphorylase (MTAP) gene deletion occurs in ~15% of solid tumors, including 15% of NSCLC, 28% of esophageal cancer, 26% of bladder cancer, and 10% of esophagogastric cancer. For tumor cells deficient in methythioadenosine phosphorylase (MTAP), Met depletion and inhibition of the key enzyme MAT2A in the Met metabolism pathway are possible therapeutic strategies. MAT2A inhibitors AG-270 and IDE-397 have demonstrated significant antitumor activity both in vitro and in animal models, and phase I clinical trials are currently underway.^240,333,334^

Currently, amino acid metabolism-targeted therapy still faces many challenges. Adipocytes and bone marrow stromal cells in the TME can promote the resistance of tumor cells to ASNase treatment by supplying Gln and cysteine to leukemia cells,^348,349^ and cancer-associated fibroblasts can secrete Asp to promote solid tumor growth.^57,350^ Many lines of evidence have found that tumor resistance is caused by cells and the external environment in which the cells are located. The efficacy of a drug also depends on its ability to reach the tumor site. When the drug fails to reach the tumor site, it fails to induce tumor cell death successfully. In addition, immune and allergic reactions to non-human enzymes can compromise therapy and harm patients.^344,351^ Therefore, a comprehensive understanding of the metabolism-dependent characteristics of various tumor types and their microenvironment is needed. Decoding the metabolic requirements of amino acids in different tissues and understanding how to target the metabolism and metabolic pathways of these amino acids is indispensable for improving the level of cancer treatment.
